# Advancing maternal and perinatal health in low- and middle-income countries: A multi-country review of policies and programmes

**DOI:** 10.3389/fgwh.2022.909991

**Published:** 2022-10-10

**Authors:** Uzma Syed, Mary V. Kinney, Ekaterine Pestvenidze, Alren O. Vandy, Karin Slowing, Janet Kayita, Alyona F. Lewis, Sartie Kenneh, Francis L. Moses, Atiya Aabroo, Ellen Thom, Qudsia Uzma, Nabila Zaka, Kim Rattana, Kannitha Cheang, Robert M. Kanke, Brigitte Kini, Jean-Bertin E. Epondo, Allisyn C. Moran

**Affiliations:** ^1^World Health Organization, Geneva, Switzerland; ^2^School of Public Health, University of the Western Cape, Bellville, South Africa; ^3^World Health Organization, Tbilisi, Georgia; ^4^World Health Organization, Freetown, Sierra Leone; ^5^Pan American Health Organization, Guatemala City, Guatemala; ^6^Ministry of Health and Sanitation, Freetown, Sierra Leone; ^7^Ministry of NHSR&C, Islamabad, Pakistan; ^8^World Health Organization, Islamabad, Pakistan; ^9^National Maternal and Child Health Centre, Phnom Penh, Cambodia; ^10^World Health Organization, Phnom Penh, Cambodia; ^11^World Health Organization, Kinshasa, Democratic Republic of Congo; ^12^Ministry of Health, Kinshasa, Democratic Republic of Congo

**Keywords:** maternal health policy and programme, country case studies, evolution of maternal health, factors influencing maternal and perinatal health, low-and-middle-income countries

## Abstract

The Sustainable Development Goals prioritize maternal mortality reduction, with a global average target of < 70 per 100,000 live births by 2030. Current pace of reduction is far short of what is needed to achieve the global target. It is estimated that globally there are 300,000 maternal deaths, 2.4 million newborn deaths and 2 million stillbirths annually. Majority of these deaths occur in low-and-middle-income countries. Global initiatives like, Ending Preventable Maternal Mortality (EPMM) and Every Newborn Action Plan (ENAP), have outlined the broad strategies for maternal and newborn health programmes. A set of coverage targets and ten milestones were launched to support low-and-middle-income countries in accelerating progress in improving maternal, perinatal and newborn health and wellbeing. WHO, UNICEF and UNFPA, undertook a scoping review to understand how country strategies evolved in different contexts over the past two decades to improve maternal survival and wellbeing, and how countries in similar settings could accelerate progress considering the changing epidemiology and demography. Case studies were conducted to inform countries in similar settings and various global initiatives. Six countries were selected based on standard criteria—Cambodia, Democratic Republic of the Congo, Georgia, Guatemala, Pakistan and Sierra Leone representing different stages of the obstetric transition. A conceptual framework, encapsulating the interrelated factors impacting maternal health outcomes, was used to organize data collection and analysis. While all six countries made remarkable progress in improving maternal and perinatal health, the pace of progress and the factors influencing the successes and challenges varied across the countries. The context, opportunities and challenges varied from country to country. Two strategic directions were identified for next steps including the need to implement and evaluate innovative service delivery models using an updated obstetric transition as an organizing framework and expanding our vision to address equity and well-being.

## Introduction

The Sustainable Development Goals (SDGs) prioritize maternal mortality reduction, with a global average maternal mortality target of <70 per 100,000 live births and a supplementary national target that no country should have a maternal mortality ratio (MMR) >140 per 100,000 live births by 2030. Targets were also set to reduce newborn mortality rate to 12 per 1,000 live births and stillbirth rate to 12 or fewer per 1,000 total births in every country (1, 2). In 2017, almost 300,000 women died due to pregnancy and childbirth with almost all deaths in low- and middle-income settings, with an average annual rate of reduction of 2.9% between 2000 and 2017 (3). Given the current pace of progress, it is unlikely the SDG target will be achieved as well as an additional burden on maternal health and well-being. For every woman who dies of pregnancy-related causes, many more will suffer from morbidity, disabilities and long-term ill-health. Additionally, as per the recent estimates, the annual newborn deaths (deaths within the firs month of birth) and stillbirths (fetal death after 28 weeks of gestation) were 2.4 million and 2 million respectively and over 40 percent of all stillbirths occurred during labor (4, 5). In order to obtain the ambitious SDG targets, there is a need to accelerate maternal and newborn health, especially in high-burden settings.

A number of initiatives are underway in advancing maternal and perinatal health and survival. Ending Preventable Maternal Mortality (EPMM), a global initiative to address maternal deaths, and Every Newborn Action Plan (ENAP), a global initiative to address stillbirths and neonatal deaths, aim to accelerate and track progress in improving maternal, perinatal and newborn health and well-being (6, 7). Many countries have initiated implementation; however, accelerated efforts are needed to further operationalize at country level toward achieving the SDGs. There is a need to review current strategies to improve maternal and perinatal survival and well-being, building on existing evidence and lessons learned and taking into consideration changing epidemiology and demography.

WHO, in collaboration with UNICEF and UNFPA, undertook six country case studies to document how strategies evolved in different contexts to improve maternal and perinatal survival and well-being. The purpose of the country case studies was to inform low-and-middle-income countries as well as to inform global initiatives, such as, EPMM and ENAP, to support country implementation.

The obstetric transition framework (8) was used to identify countries at different stages of the transition process, with different MMR level and contexts (Box 1), in an effort to document lessons from countries to understand what worked and what did not in addressing maternal health. This would provide information to countries with similar contexts to adopt successful strategies to accelerate progress and move to the next stage.

Box 1 **Obstetric transition.**

The obstetric transition framework is a phenomenon first described in 2014 by Souza and colleagues, grouping countries at five different levels of maternal mortality and describing potential programmatic interventions in each stage to facilitate transitioning to higher stages with lower levels of mortality. [“Obstetric transition: the pathway towards ending preventable maternal deaths” (8)]. However, it may be mentioned that this is a simplified way of describing the transition while, in reality, countries transition in varying pathways according to their own contexts and not always in a linear fashion. The characteristics of various stages of Obstetric Transition are summarized below:

**Table:** 

**Stage**	**MMR**	**Characteristics**
I	>1000	• Poor access to services • Most births in the community • High fertility rates • Death from communicable diseases
II	300-999	• High fertility • Access to care scaled up and women are starting to seek care
III	50-299	• Access still a challenge but women are seeking services • Quality of care is challenge • Training of health workers, supplies and quality of care are issues
IV	<50	• Fertility low • Indirect causes start to be an issue • Overmedicalization begins
V	All avoidable maternal deaths prevented	• Fertility low • Indirect causes and NCDs main causes; • Challenge is ensuring equity especially vulnerable groups

The objectives of the country case studies were to:

- Describe the trends in maternal mortality, stillbirth rates, coverage and other key outcomes between 2000 and 2019;
- Document the factors that may have contributed to maternal and perinatal survival and well-being including health systems factors and contextual/ political factors;
- Examine national policy, health system, and programme changes to identify important events that may have contributed to scale up of maternal and perinatal health;
- Document how health system is organized to better understand how maternal healthcare is provided.

## Methods

A Steering Committee was convened to oversee the research. The members of the Steering Committee included representatives from ministries of health, academic and research institutes, professional associations, maternal and newborn health experts from various geographies. The country case studies were presented to WHO's Public Health Ethics Consultative Group and feedback was incorporated.

### Selection of countries

Countries were selected for the case studies based on following criteria: (1) varying rates of progress in maternal mortality reduction; (2) representation across the WHO regions; and (3) stage of Obstetric Transition.

The selected countries (Table 1) included Cambodia, Democratic Republic of the Congo (DRC), Georgia, Guatemala, Pakistan and Sierra Leone, and represented five out of six WHO regions.

**Table 1:** Maternal health scoping review case studies countries.

**Obstetric** **transition** **stage**	**Country**	**MMR 2000** **(per 100,000** **live births)**	**MMR 2017** **(per 100,000** **live births)**	**Average** **annual rate of** **reduction** **2000–2017**	**Number of** **maternal** **deaths in 2017**	**Stillbirth rate** **in 2019 (per** **1,000 total** **births)**
GLOBAL	NA	342	211	2.9%	295,000	13.9
Stage I (MMR >1,000)	Sierra Leone	2,480	1,120	4.7%	2,900	27.3
Stage II (MMR 300–999)	DRC	739	378	2.8%	16,000	27.2
Stage III (MMR 50–299)	Guatemala	161	95	3.1%	400	12.7
	Pakistan	286	140	4.2%	8,300	30.6
	Cambodia	488	160	6.6%	590	12.4
Stage IV (MMR <50)	Georgia	31	25	1.3%	14	5.7

Data sources: WHO, UNICEF, UNFPA, World Bank, UNDP, Trends in maternal mortality: 2000 to 2017. Geneva: World Health Organization; 2019.

UN Inter-agency Group for Child Mortality Estimation, A Neglected Tragedy, The Global Burden of Stillbirths, 2020.

### The conceptual framework

**Figure 1:**
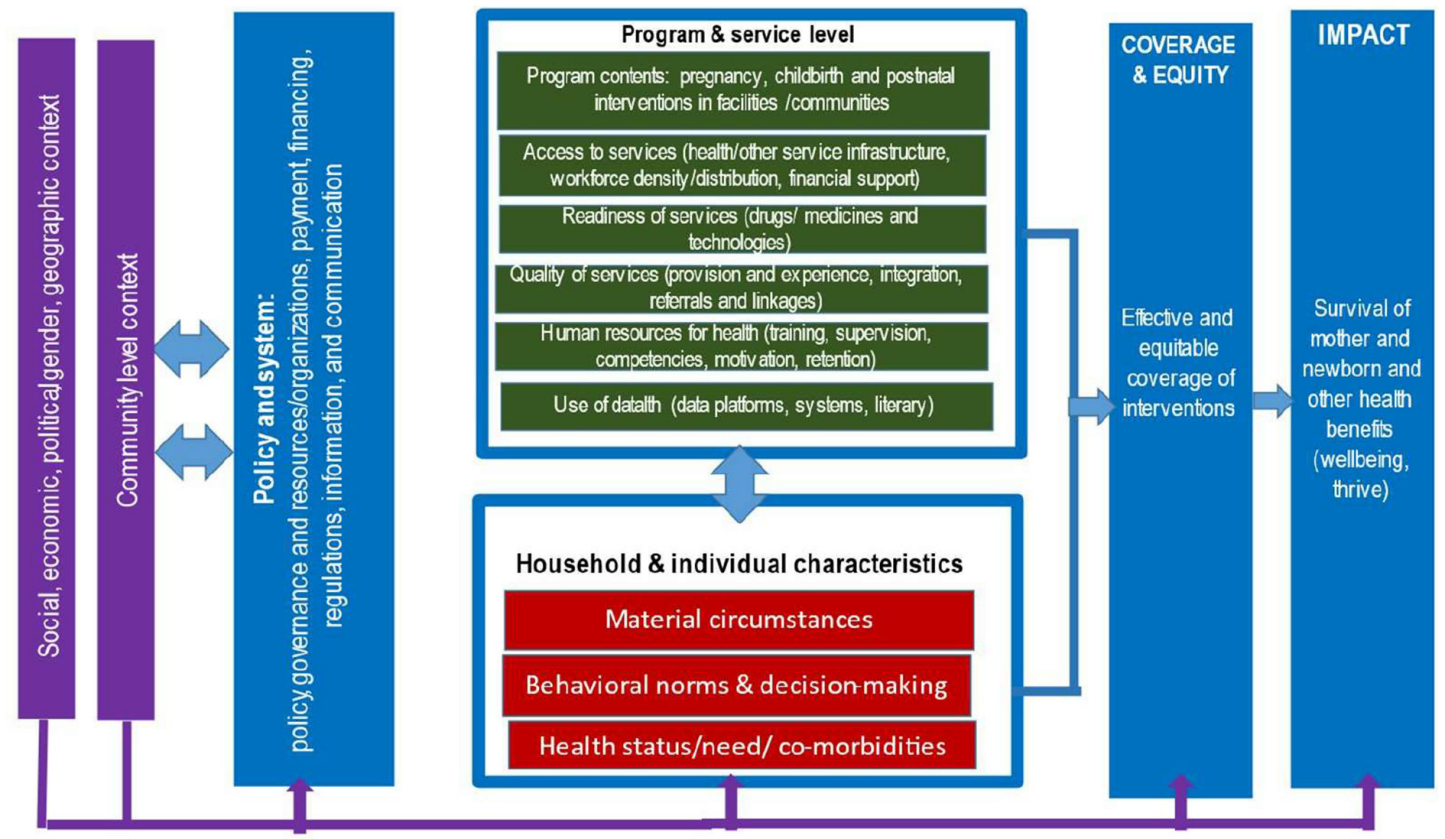
Conceptual framework for maternal health scoping review.

The case studies were organized based on a health system conceptual framework (Figure 1) to assess the process and impact of various components on maternal and perinatal health outcomes over the past two decades. The framework aims to encapsulate the range of interrelated factors impacting maternal and newborn health (MNH), including the non-health contextual influences, health policy and system levers, the programme and service level components as well as individual and household characteristics leading to the provision and coverage of interventions toward the impact on maternal and perinatal health and wellbeing. The framework aligns with the conceptual framework developed by the Exemplars Maternal and Newborn Health study (9) with some variations. Standard tools and data collection instruments were developed to assess each of the objectives. The conceptualization of the framework, data collection tools, and selection of countries took place in 2019.

### Data collection

The researchers worked with the regional and country offices to identify local consultants in each country, who had experience in researching and engaging in MNH. The consultants led the desk review, organized stakeholder consultations and engaged in the cross-country consultation workshop with representation of key stakeholders. An orientation was conducted with each consultant and the respective WHO regional and country office colleagues. Due to the COVID-19 pandemic, each orientation session was conducted in a virtual format, and the researchers provided continuous follow up and remote support throughout the data collection process.

Data sources, tools and analyses varied for each of the four case studies objectives. The methods for data collection can be grouped as desk reviews, meetings with stakeholders and cross-country consultation workshop.

### Desk reviews

The local consultants conducted desk reviews and collected data on key outcomes related to maternal health as well as on changes in policies and programmes over time and to document how maternal health services are delivered at various levels of care. National policies, strategies and plans, as well as program documents and reports relevant to maternal and perinatal health were reviewed and incorporated into the data collection templates. The documents, including the peer-reviewed and gray literature, were suggested by the country stakeholders and were also identified through structured searches including hand-searching. In some countries, interviews with key stakeholders were conducted either by phone or in-person, depending on the COVID-19 pandemic restrictions.

#### Trends in maternal mortality, stillbirth rates, coverage of services and key outcomes

Relevant data on maternal mortality ratios, morbidity, stillbirth rates, coverage and financing between 2000 and 2019 were collected applying a systematic process. Definitions of the mortality indicators are provided in Box 2. Data were compiled from global estimation processes as well as from national statistics, nationally-representative household surveys and administrative data and surveillance systems.

Box 2 **Key definitions (source: https://www.who.int/data/gho/indicator).**

Maternal Mortality Ratio (MMR): The maternal mortality ratio is defined as the number of maternal deaths during a given time period per 100,000 live births during the same time period.

Newborn Mortality Rate (NMR): Number of deaths during the first 28 completed days of life per 1000 live births in a given year or other period.

Stillbirth rate (SBR): The stillbirth rate is defined as the number of babies born with no signs of life at 28 weeks or more of gestation, per 1,000 total births.

Perinatal death rate: All stillbirths plus neonatal deaths through the first 7 completed days after birth (https://www.who.int/publications/i/item/9789241511223).

#### Policy and programme timeline

A Policy and Program Timeline template was developed to document factors that may have contributed to maternal and perinatal survival from 2000 to 2019 including health systems factors, contextual/political factors, and programme changes. The data for template were collected through desk reviews and interviews with key stakeholder. The idea for the Timeline initially came from a figure in The Lancet's Alma Ata Series, and has been adapted by others, including Saving Newborn Lives (10) and Countdown to 2015 for country case studies (11).

#### Organization of service delivery including networks of care

To understand maternal and newborn service organization and linkages across different levels of the health system, including the private sector, a second tool was adapted from WHO recommended interventions for improving maternal and newborn health—Integrated management of pregnancy and childbirth (2009) (12). The idea for mapping of service delivery initially came from the Maternal Health Lancet Series (13), in an effort to inform the ongoing re-visioning of Emergency Obstetric Care. Examples of functional networks of care was also included.

### Meetings with stakeholders

After completion of the templates, meetings with stakeholders were convened to review and discuss the data from the templates on Policy and Programme Timeline and Organization of Service Delivery and come to consensus on key lessons learned. Stakeholders included representatives from Ministries of Health, UN agencies, professional organizations, non-governmental organizations, academics and civil society groups. Depending on COVID-19 restrictions, some meetings were in person while others were virtual. All data were collected in 2020.

### Virtual cross-country consultation

A virtual workshop was held in August 2021 to discuss the findings with relevant country focal persons, stakeholders and maternal and newborn health experts, including the ministries of health representatives, engaged in the case studies. A total of 51 participants attended the 2-day virtual workshop. The objectives of the workshop were to:

- Exchange lessons from diverse approaches applied by countries at various stages of obstetric transition
- Jointly identify lessons learned and implications for countries with similar contexts toward improving maternal and perinatal health.

Country-specific lessons were shared and discussed at the workshop. Based on the consultation, lessons were updated and refined. A set of key recommendations were also drawn from the workshop.

## Data analysis

Trends in mortality, stillbirths, and coverage were analyzed using MS Office software. Descriptive analyses of the WHO SRMNCAH Policy Survey were conducted using STATA software and shared with country-level stakeholders. For each country, a qualitative thematic analysis was conducted based on the conceptual framework (Figure 1). Data were compiled from the desk review, interviews, and completed templates and organized according to the analysis framework below and compiled in a table (see Annex I and II in Supplementary materials 1, 2). The components of the framework used for data analysis included the contextual factors external to the health sector, policy and system levers, programme and service levels (Table 2).

**Table 2:** Analysis framework.

**Component**	**Description of component**
**Non-health context**	
Social status of women and girls	Women's rights, early marriage, gender-based violence
Response/ Resilience	Humanitarian crisis, natural disasters, population displacement, etc. influencing service delivery and access (excluding COVID-19)
**Policy and system**	
Policies	National/sub-national policies maternal and newborn (MNH) strategies, plans
Maternal and Newborn Targets	National targets for maternal and newborn mortality and prevention of stillbirths
Leadership	Political will, individual champions, national technical working groups/committees
Financing	Financial resources including government, private, domestic, external, out-of-pocket payment, pro-poor schemes
**Programme content**	
System of care	Levels of care, organization of services
Maternal and perinatal services	Services available as described in the national guidelines
Networks of care	Networks of facilities and health care providers
**Programme implementation**	
Access to services	Availability of services, workforce density/distribution
Quality of care	Provision and experience of quality and respectful care
Data	Data platforms, systems, use of data for decision making
Health care workers	Policies/plans for maternal and newborn providers both in public and private sectors, training, competencies, supervision, motivation
Private sector	Role of private sector including regulation and engagement with public sector
Community engagement	Involvement of communities, women and families in maternal and perinatal care

Three researchers (Syed, Kinney and Moran) first looked at the findings in each country and then synthesized them across the countries. Basic background characteristics such as population, fertility rate, health policy cycle, national targets, and health expenditure were included for each country (Table 3). All relevant information was manually extracted from the country reports and templates and organized by country into a table. The cross-country data analysis was performed for each of the components and classified into successes and challenges. Lessons were drawn for each component from the findings across the six countries. The findings were summarized and shared with country teams for review and feedback in order to develop consensus. After their inputs were incorporated, a revised version was circulated prior to the cross-country consultation.

**Table 3:** Background characteristics by country.

**Country**	**Georgia**	**Cambodia**	**Guatemala**	**Pakistan**	**Sierra Leone**	**Democratic** **Republic of** **the Congo**
**Obstetric transition**	Stage IV	Stage III	Stage III	Stage III	Stage I	Stage II
**Population** **(****14****)**	Total: 3.97 million Total births (2021): 50,198	Total:16.95 million Total births (2021): 357,220	Total: 18.25 million Total births (2021): 428,935	Total: 225.2 million Total births (2021): 6,049,547	Total: 8.14 million Total births (2021): 259,319	Total: 92.4 million Total births (2021): 3,663,456
**Total fertility rate (live birth/woman)**	TFR: 2 LB/woman	TFR: 2.4 LB/woman	TFR: 2.7 LB/woman indigenous: ~44% (IWGIA)	TFR: 3.3 LB/woman	TFR: 4 LB/woman	TFR: 5.6 LB/woman
**Female literacy rate** **(****15****)**	99% (2019)	75% (2015)	77% (2018)	46% (2019)	35% (2018)	66% (2016)
**Health system** ^**a**^	Centralized	Centralized	Decentralized	Devolved	Centralized	Centralized
**Policy cycle**	National MNH strategy 2017–2030 and Action Plan (2017–19)	NSDP 2019–2023; HSP 4 2021–2030 (under development)	5-year plans for reduction of maternal and neonatal mortality rates (2015–2020; 2020–2025)	10-yr Dev. Plan (2025); Health Vision 2016–2025; 5-yr Action Plan (2019–2023)	National Health Sector Strategic Plan, 2017–2021; RMNCAH Strategy (2017–2021)	5-year National Health Development Plans (2011–2015; 2016–2020; reframed 2019–2022)
**Proportion of births by type of health personnel**	OBGYN/doctors: 100% (NCDC 2020)	Midwife: 70.5% Doc: 15.3% Traditional Birth Attendants (TBA): 10.7% (CDHS 2014)	Hospital doctor: 45% Nurse/mw: 3.8% Unskilled Birth Attendant (non-SBA): 33.5% (ENSMI 2014)	Doctors: 60% TBA: 24% (PDHS 2018)	PHU Nurse/mw: 53.1% Hosp nurse/mw: 25.7% Hosp doc: 3.7% non-SBA: 12% (DHS 2019)	Nurse/male nurse: 40.3% Midwife: 34.4% Doc: 10.4% (MICS 2018)
**Private sector (% of facility births)**	100%	14.3% (2014); 17% of total inst. deliveries (83.2%)	Private: 8.5% MSPAS: 48.3% IGSS: 8.2%	42.6% (2019); 59% of total (71.1%)	2.2% (2019); 2.6% of total (83.4%)	15.4% (2014); 19% of total (79.9%)
**Health expenditure:** Total Health expenditure (THE) as % of GDP and Out of pocket spending (OOPS) as % of THE	THE: 11.4% in 2000, 21.3% in 2010, 40.8% In 2019 OOPS: 81% in 2000; 47% in 2019	THE: 6.5% in 2000, 6.9% in 2010, 6% in 2019 OOPS: 68.6% in 2000, 51.9% in 2010, 64.3% in 2019	THE: 5.4% in 2000, 6.1% in 2010, 6.2% in 2019 OOPS: 59% in 2000, 59% in 2010, 56% in 2019	THE: 35% in 2000, 22% in 2010, 32% in 2019 OOPS: 61.5% in 2000, 70% in 2010, 53.8% in 2019	THE: 11.5% in 2000, 10.9% in 2010, 8.7% in 2019 OOPS: 75.6% in 2000, 64% in 2010, 55% in 2019	THE: 4.41% in 2008, 4.14% in 2010, 3.65% In 2019 OOPS: 43.25% in 2008, 36.91% in 2010, 44.53% in 2019

Data sources: WHO Policy country profile; https://platform.who.int/data/maternal-newborn-child-adolescent-aging/static-visualizations/policy-country-profile.

The World Bank data; https://data.worldbank.org/indicator/SE.ADT.LITR.FE.ZS.

National Center for Disease Control and Public Health (NCDC), Statistical Yearbook Georgia, 2020. https://www.ncdc.ge/#/pages/file/06d0d272-e413-4ae8-914a-4a0a42e98d53.

National Health Account (NHA) data, apps.who.int/nha/database.

Notes: a. Decentralized and Devolved systems: The OECD defines decentralization as measures that transfer a range of powers, responsibilities and resources from central government to subnational governments, defined as legal entities elected by universal suffrage and having some degree of autonomy. Devolution is a subcategory of the decentralization concept. It is a stronger form of decentralization as it consists of the transfer of powers from the central government to lower-level autonomous governments, which are legally constituted as separate levels of government (16).

## Results

### Background characteristics of the case studies countries

The present characteristics, relevant to maternal health policies and programmes, were documented for each country. Table 3 includes the characteristics, such as the present status in Obstetric Transition framework, population size, total fertility rate, female literacy rate, type of health system, policy cycle, health personnel in and proportion of private sector in childbirth care and health expenditure [total health expenditure (THE) as part of Gross Domestic Product (GDP) and out-of-pocket spending (OOPS)].

### Impact and coverage

**Figure 2:**
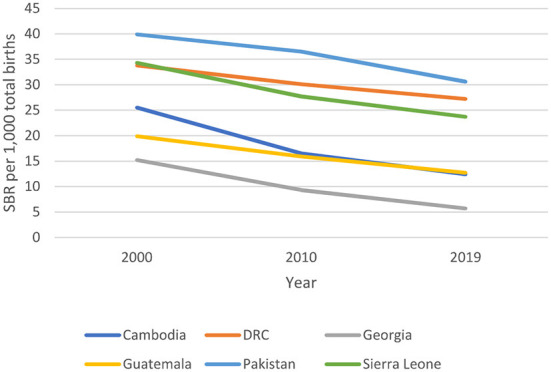
Trends in Maternal Mortality Ratio, 2000-2017 (3).

**Figure 3:**
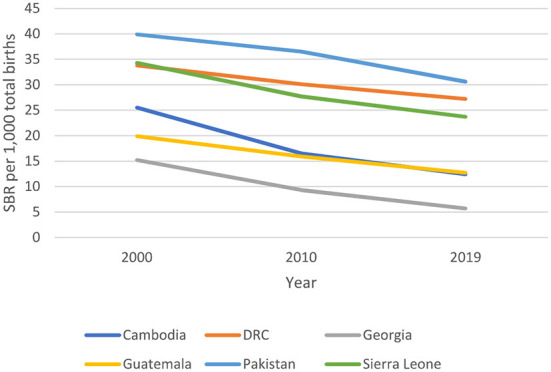
Trends in Stillbirth Rate (SBR) 2000-2019 (5).

**Figure 4:**
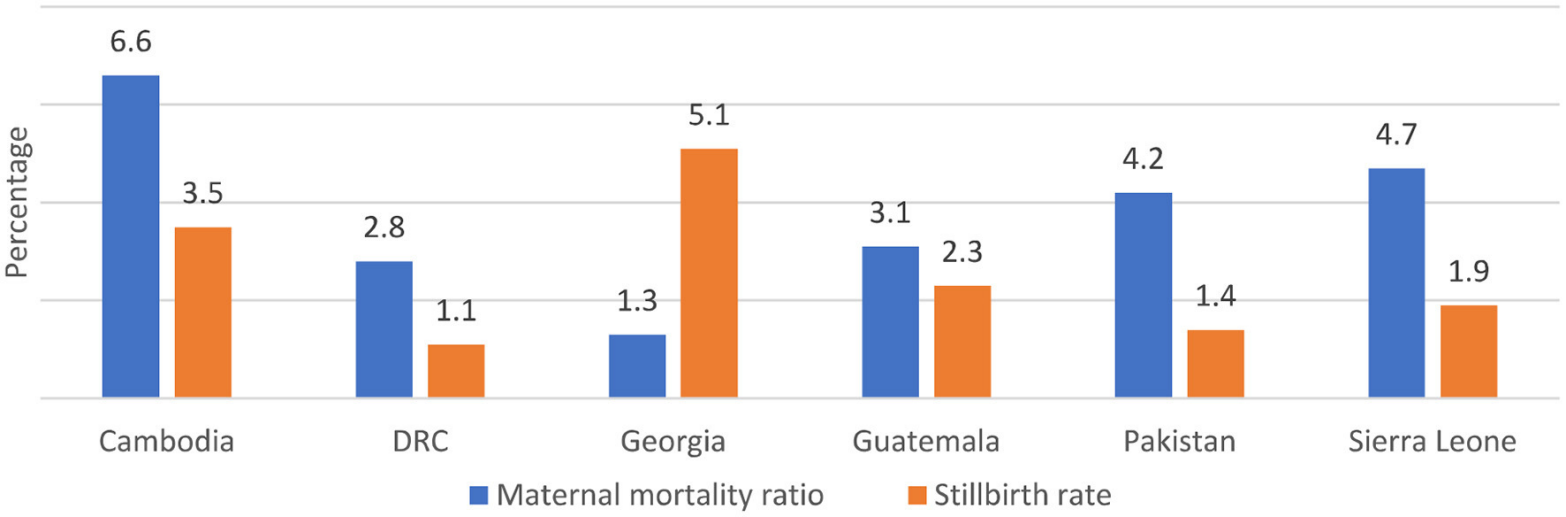
Annual rates of change in MMR and Stillbirth rate in six countries (3, 5).

**Figure 5:**
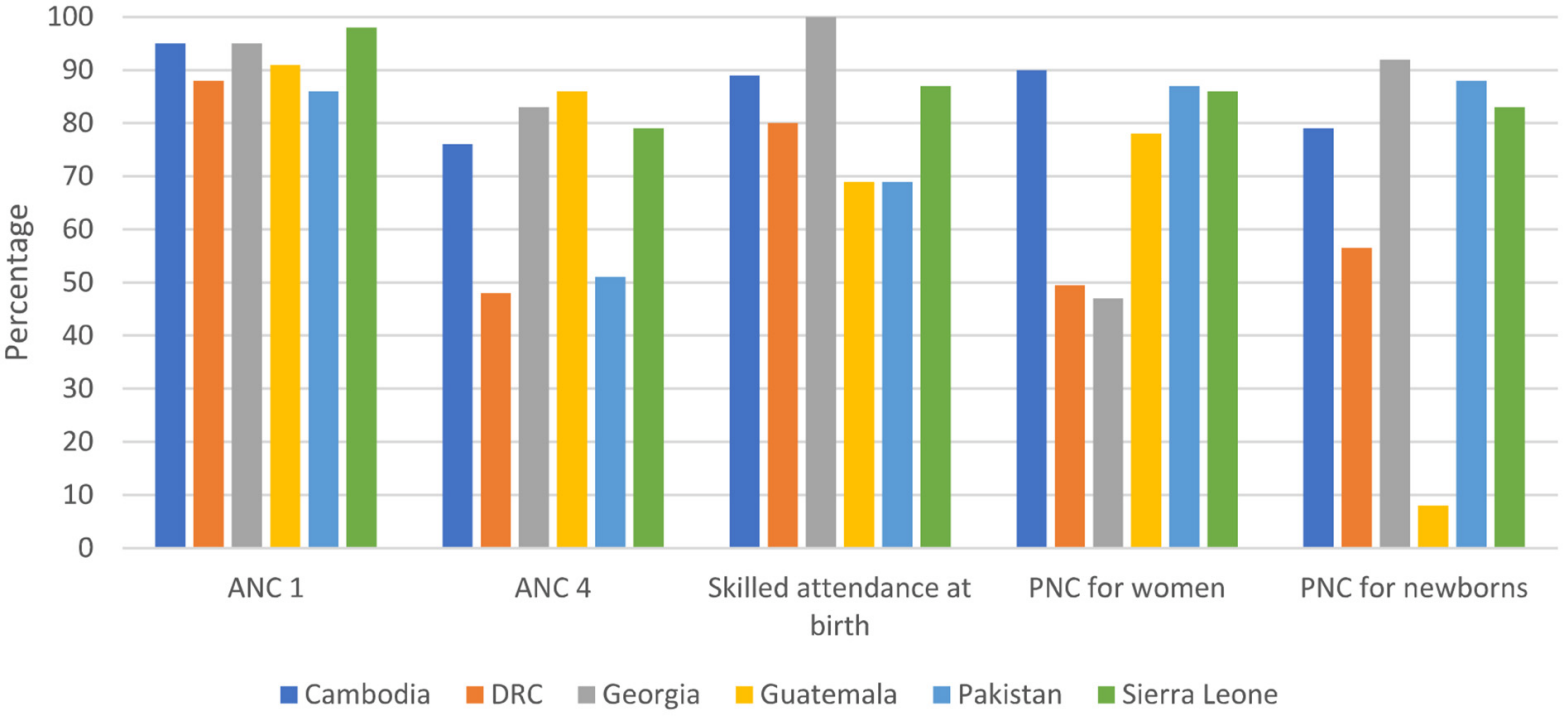
Coverage of essential interventions across the continuum of care (Sources: Cambodia DHS 2014, DRC MICS 2018, Georgia NCDC 2019, Guatemala ENSMI 2015, Pakistan DHS 2018, Sierra Leone DHS 2019).

Maternal mortality ratio and stillbirth rates declined in all countries between 2000 and 2017 (Figure 2) and 2000 to 2019 (Figure 3) respectively, with varying average annual rates (Figure 4). There was a great variation in coverage of essential maternal and perinatal health services (Figure 5).

**Figure 6:**
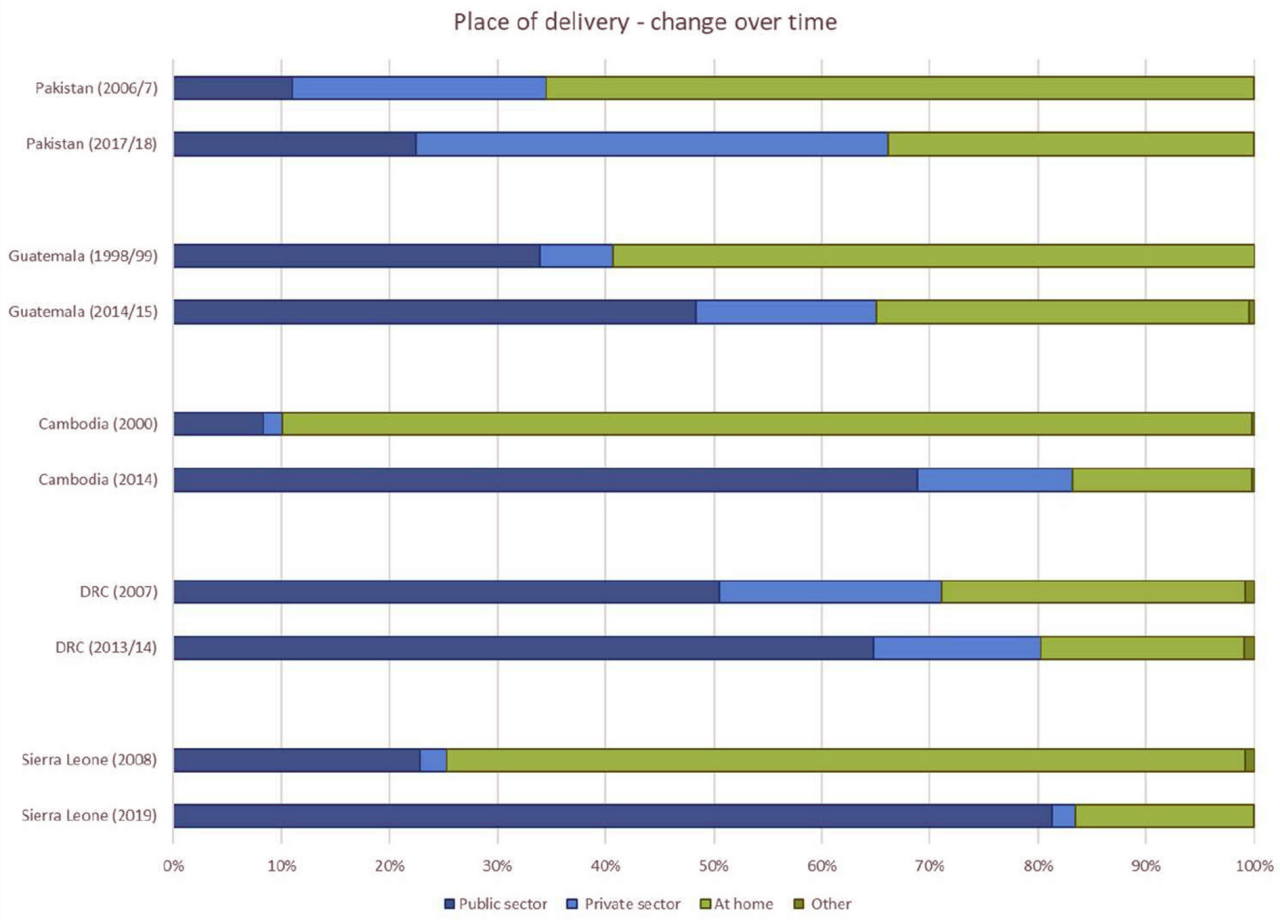
Place of birth over time for five countries (Sources: Data from Demographic and Health Surveys in countries, Statcompiler accessed on 11 October 2021).

**Figure 7:**
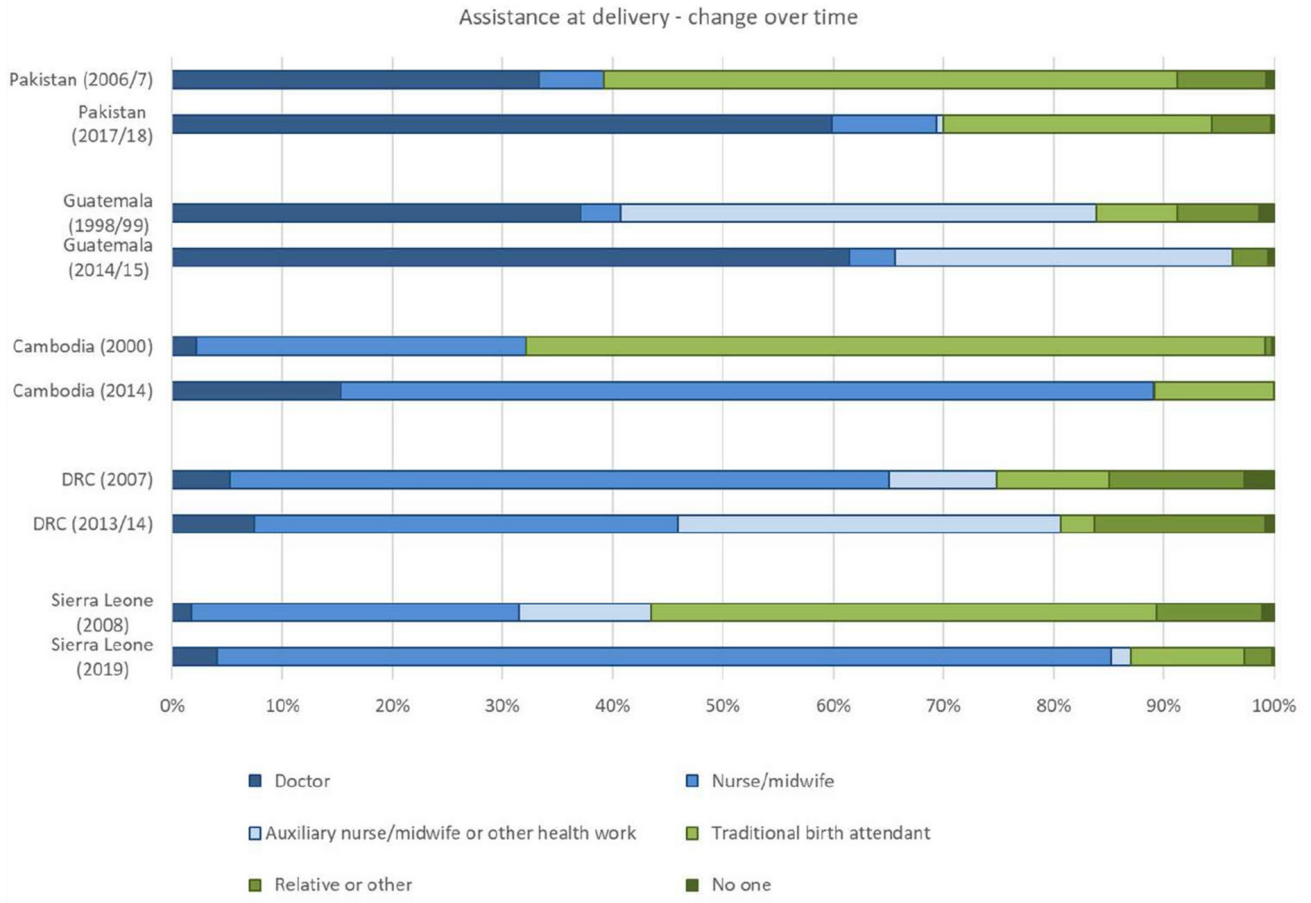
Assistance at birth over time for five countries (Sources: Data from Demographic and Health Surveys in countries, Statcompiler accessed on 11 October 2021).

Figures 6, 7 display the place of birth and assistance at birth over time for the five countries with available data from Demographic and Health Surveys (DHS), i.e., Cambodia, DRC, Guatemala, Pakistan and Sierra Leone. All countries experienced an increase in the number of births taking place at health facilities (public or private) and an increase in skilled attendance at delivery. Some countries have seen remarkable change in place of delivery, such as Sierra Leone and Cambodia.

### Success factors, challenges and lessons learned

#### Non-health context

The data provided an understanding of various settings considering changes in contextual determinants such as social status of women and girls, humanitarian and fragile contexts over the past two decades. In Cambodia, where MMR reduced at an annual average rate of 6.6%, had a sustained approach in improving the laws and regulations related to women's and girls' rights and education. This was also noted for Georgia. Policymakers prioritized narrowing of the gender gap in primary education in Cambodia (14). Social status of women and girls varied at subnational level in Guatemala and Pakistan, the countries in stage III of the obstetric transition. In Sierra Leone, a stage I country with very high mortality and high rate of gender-based violence, had low female literacy rate [35% (12)].

All countries reported a variety of emergency situations that impacted access and delivery of essential maternal and newborn health services. This included humanitarian crises, conflicts, epidemics and natural disasters. For a number of years following the disasters or conflicts, countries faced constant sub-national level migration of displaced populations. Our analysis showed that maternal and perinatal health were not prioritized at the service delivery level. While such emergencies drew global attention and funding, planning and resource allocation addressed only the short-term needs.

#### Policy and system

National policies and plans were promptly updated to include evidence-based guidelines, standards, and interventions. However, plans were not always costed which resulted in weak implementation. In addition, countries with devolved administrative structures, such as Pakistan, had additional challenges in terms of consistency in implementation since they were largely driven by the sub-national authorities. Provinces with strong stewardship successfully adapted the policies into practice. Stakeholders in Cambodia attributed the maternal health outcomes to the alignment of strategies across multiple sectors. The private sector, often playing an important role in maternal and newborn healthcare, was not consistently engaged in planning implementation. Having Maternal Health incorporated in the broader development agenda helped to engage non-health sectors in Cambodia, Georgia and Guatemala toward improving maternal health outcomes.

Countries set and revised targets during the development of the health sector plans and/or national development plans. Maternal mortality and neonatal mortality targets were more commonly established than the target for stillbirth.

In the case studies, it was clear that sustained political will was essential for progress in maternal and perinatal health. Political champions were very effective but not sustainable with change in government. National policies and implementation strategies got substantially and frequently modified with the change of the political government. Shifts in governments impacted service delivery and resource allocation. There was some evidence that the national technical working group was able to maintain momentum even during shifts in political leadership, as in Guatemala.

**Figure 8:**
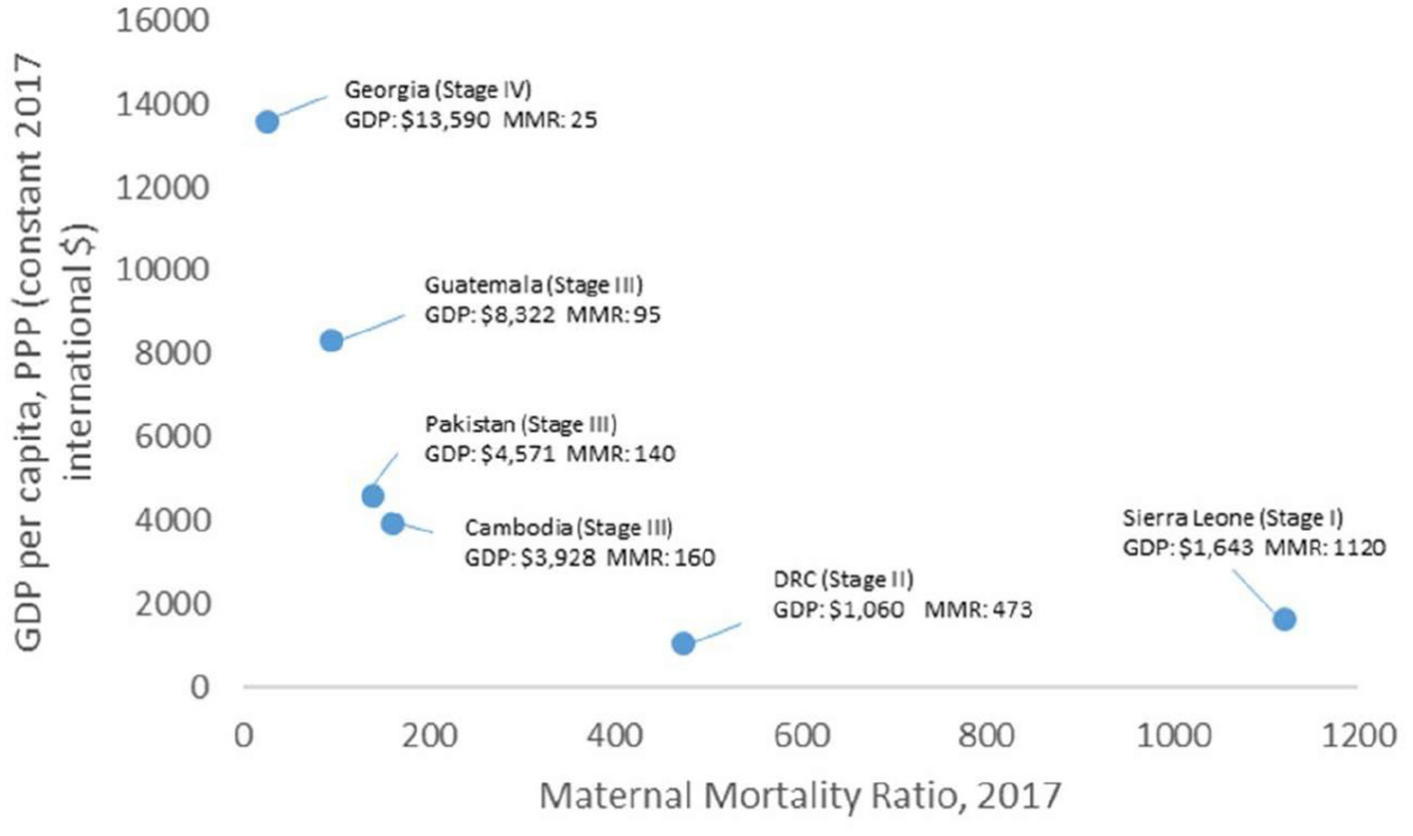
Comparison of the MMR data against GDP in six countries [Sources: World Bank (GDP) and World Health Organization (MMR)].

We also examined trends in financing. Countries with lower levels of GDP also had higher maternal mortality ratios (Figure 8). Total Health Expenditure (THE) remained low across all countries (Table 3) except for Georgia where THE increased from 11% in 2000 to 41% in 2019. High out-of-pocket spending for health remained a challenge across countries. Large-scale programmes, often supported by external donors, are time-bound and resource allocation suffered from withdrawal of donor support. Innovative financial schemes were often supported by external donors and did not sustain over time.

**Figure 9:**
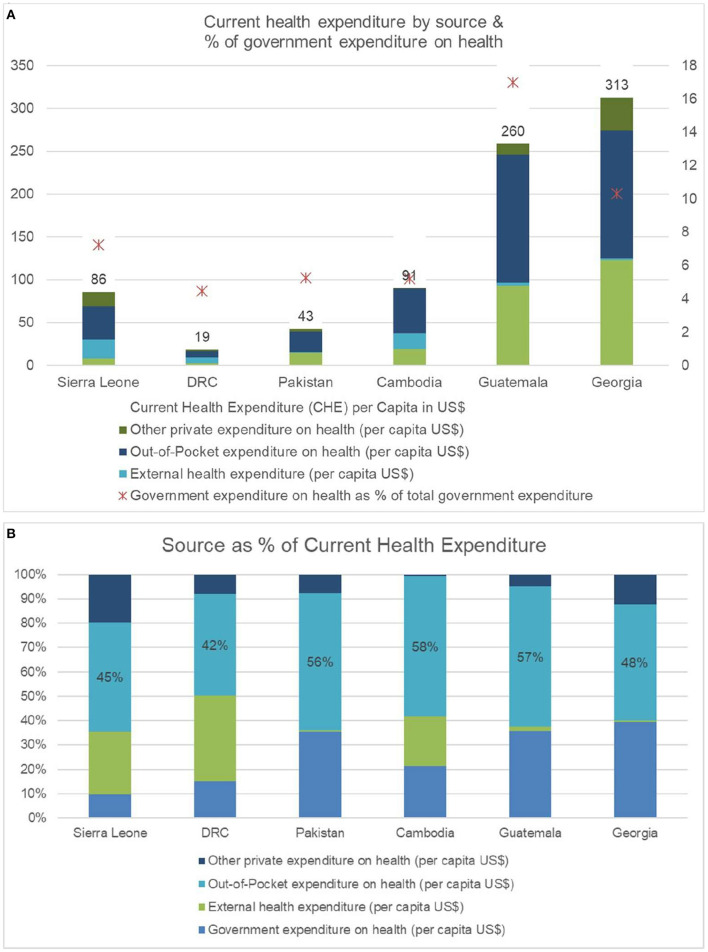
**(A)**: Total health expenditure (THE) by source across the six countries (Source: Global Health Expenditure Database accessed on October 6, 2021). **(B)**: Proportion of funding by source across six countries. (Source: Global Health Expenditure Database accessed on October 6, 2021).

Figure 9A shows the total health expenditure by source across the six countries. Figure 9B shows the proportion of funding by source. While the total health expenditure is variable across the countries, the proportion of out-of-pocket expenditure ranges from 42 to 58%. Only one country exceeded 15% of government expenditure on health as a proportion of total government expenditure.

#### Programme content

We documented the organization of the system (i.e., levels of care, organization of services) as described in national guidelines to understand how maternal health services are organized across different levels. In all six countries, there were clear structured levels of care outlined in national policies, plans and strategies. However, the definition of each level was country-specific and did not always follow global definitions or recommendations. In addition, there were limitations in terms of functional referral systems as well as linkages with other disease programmes (specifically non-communicable and infectious disease). While the first levels of care were mostly overburdened, there were limitations in competent human resources as well as essential medical equipment to provide the required services. In Cambodia, the midwife coordination alliance team (MCAT) facilitated maintenance of providers' skills and supervision but often funding was inadequate to convene meetings. Local health committees, such as, Consejos Comunitarios de Desarrollo (COCODEs) and networks of community health facilitators in Guatemala served as important platforms for the community but were not linked to the formal system and did not sustain over time.

MNH services were not consistent or linked across levels of care. High volume facilities with limited workforce often could not practice quality and respectful care. Management of morbidity was sometimes inadequate and/or inconsistent in the protocols. Some important morbidities, such as infections, anemia, fistula and maternal mental health including postpartum depression, were not adequately included in the guidelines.

#### Programme implementation

Access to services, quality of services, data systems and human resources were documented to understand the changes in programme implementation.

**Figure 10:**
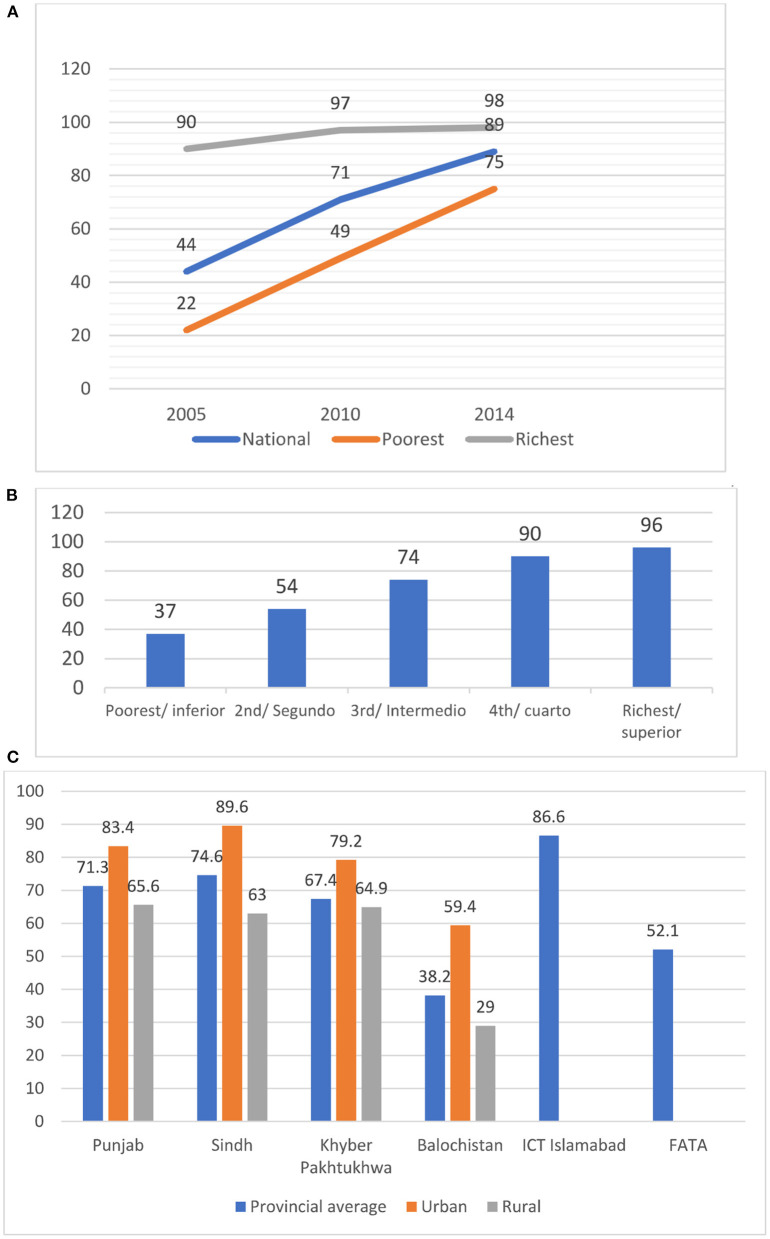
Disparities in coverage of skilled heath personnel at birth. **(A)**: Trends in inequity of skilled health personnel at birth in Cambodia (source: Cambodia Demographic and Health Surveys). **(B)**: Inequity in skilled health personnel at birth in Guatemala (source: Guatemala Encuesta Nacional de Salud Materno Infantil, 2014–2015). **(C)**: Provincial disparities in skilled health personnel at birth in Pakistan (source: Pakistan Demographic and Health Survey 2017–2018).

Surveys demonstrated in-country disparities in access to essential services across various population groups, provinces, and income groups. Coverage of essential interventions varied across the wealth quintiles, urban and rural populations, as well as among ethnic groups. Country-specific trends in inequity across the poorest and the richest quintile and also across sub-national/provincial level for skilled health personnel at birth are shown in Figures 10A–C.

Inequity in services was often not reported routinely. Although there was evidence that financial incentives might improve access to and use of some services (such as facility-based birth), these incentive schemes were often supported by donor funded programmes and were not feasible to sustain in the long run without government budget allocation. Expansion of the health equity funds (i.e., availability of no-cost health care for poor people) in Cambodia was a contributing factor in significant reduction of maternal mortality (17). Regionalization of service delivery, the example from Georgia, strategically created stratified health care facilities according to their capacity and geographic location for providing health services and linked clinics with limited capacity with stronger facilities. This resulted in improvement in level-appropriate care (Box 3).

Box 3 **Regionalization of maternal and neonatal health services in Georgia.**

In 2015, a data-driven package of reforms was introduced to improve maternal and infant health outcomes (Order N01-2n) (18) in Georgia. It envisioned creation of a comprehensive, coordinated and geographically structured system of stratified health care facilities according to their individual capacity and geographic location and provision of risk-appropriate perinatal care to all mothers and infants. (ref: https://www.euro.who.int/__data/assets/pdf_file/0008/374615/hit-georgia-eng.pdf ).

The reform, with support from United States Agency for International Development and John Snow Inc., was piloted in Imereti, Racha-Lechkhumi & Kvemo Svaneti Regions. The reform package included: a) reorganization of perinatal care service delivery, merging some of the clinics with limited capacity and low volume of deliveries to the stronger facilities with perinatal services; b) strengthening infrastructure and equipping of perinatal service providers in line with requirements of designated level of care; c) strengthening human resource capacity; d) improving transport/referral system operation. In 2016, the initiative was scaled up nationally by the Ministry of Labour, Health and Social Affairs (MoLSHA) with support from UNICEF, UNFPA, World Vision, Rotary Club Tbilisi International. The initiative led to:

100 % of perinatal facilities have assigned level of obstetrics and neonatal care according to national structure

22 (out of total 106) perinatal facilities with limited capabilities closed down

23 facilities upgraded to higher level of care

100% facilities strengthened human resource capacity

100% facilities strengthened infrastructure and equipment

95% facilities provide level-appropriate care

30% reduced newborn referral

Perinatal mortality reduced from 13.4 in 2015 to 11.7/1000 birth in 2018

Maternal mortality reduced from 32.2 in 2015 to 27.4/100 000 livebirth in 2018 (19).

Stakeholders reported that the adoption of quality-of-care policies, both for provision and experience of care, was a critical measure undertaken by countries for improving maternal and perinatal outcomes. However, policy uptake did not always link to implementation. Stakeholders stated that training of providers did not always link to improved quality of services. Providers were often overburdened and not adequately equipped and supervised to use their skills. All countries have had increased cesarean section rates over the years, especially among the wealthy quintiles. Lack of adequate data on maternal and newborn morbidity in the routine health information system and across facility registers was a barrier in understanding care-seeking and management of sick mothers and newborns. Strong regulation and monitoring of the private sector by the government helped improve accountability and quality of care in Georgia.

Availability of quality data is key to addressing implementation issues. Transitioning to digitalised data systems helped access to real time data, such as DHIS2 in countries like DRC and Georgia. There were limited or no real time data around quality of care, equity and morbidity in routine health information system. Georgia experience showed that having routine data record and report on morbidities helped improve preparedness and management at facilities. Sub-national level data availability and improved capacity in data use improved planning and designing efficient strategies at local level in Cambodia and DRC. Routine data systems did not always include data from private providers or private facilities. Adoption of the Maternal and Perinatal Death Surveillance and Response guideline to national programme at scale showed improvement in reporting and investigating the causes of deaths. We determined through stakeholder consultation that the response mechanism at facility level was not well established and the civil registration systems were weak with variations within and between countries.

**Figure 11:**
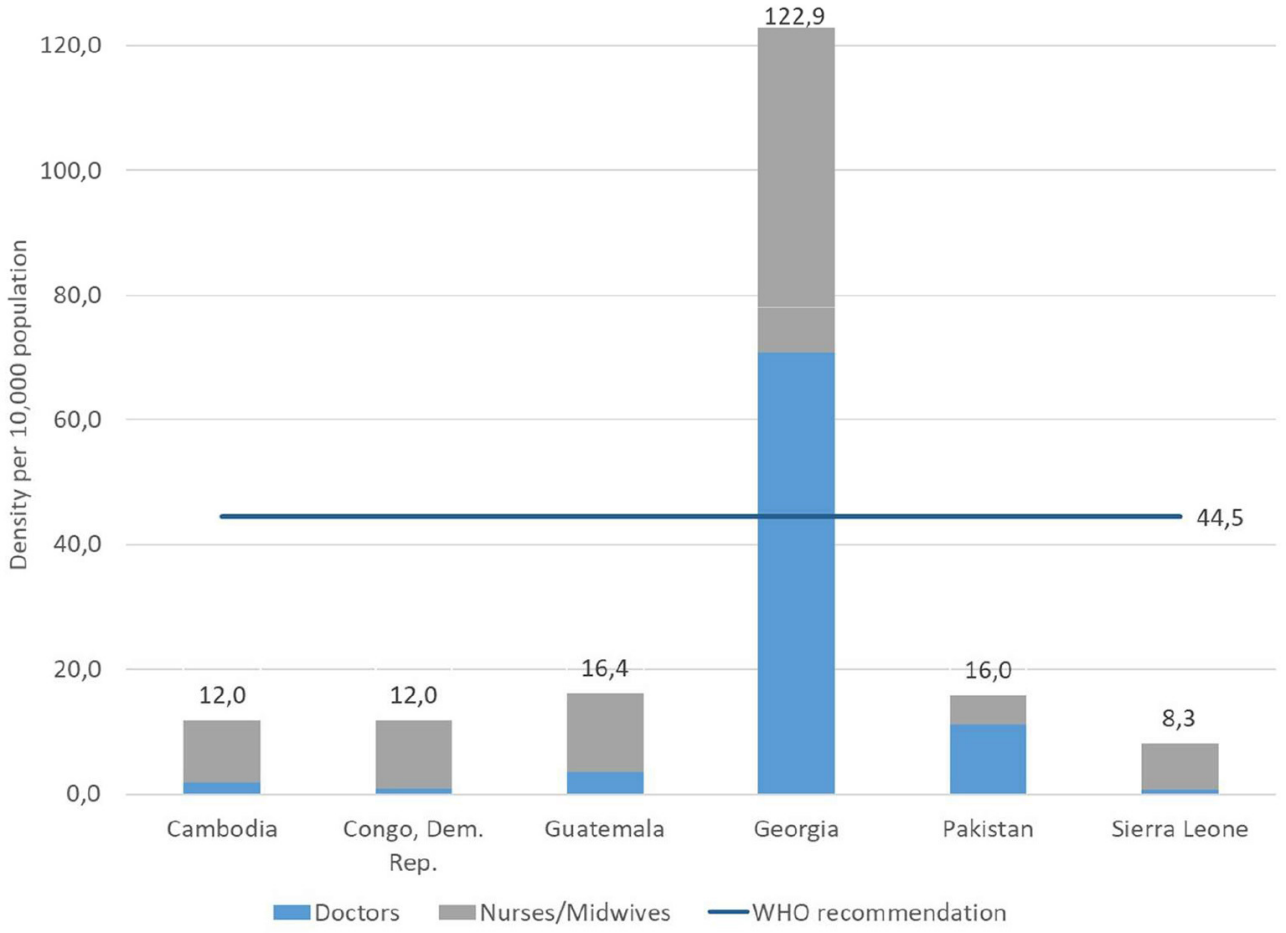
Density of skilled health professional: doctors and nurse/midwives (per 10,000 population) (Source: Global Health Observatory data repository accessed October 05, 2021).

In all countries, there were challenges around adequate equitable distribution of health care workers as well as concerns around competencies, supervision, motivation and retention of providers. Figure 11 shows the density of skilled health personnel were way below the WHO recommendation in five out of six case studies countries.

Definitions and training varied across countries for different cadres of maternal and newborn health providers (e.g., nurse-midwives, community midwives, trained traditional midwives etc.). Policies and regulations elevating the social status and acceptance of health workers, especially serving at the primary and community levels, improved motivation and performance. Stakeholders raised that the roles were not often clear at service delivery points and discrimination was also evident among the providers. Deployed midwives were not recognized or could not perform their roles in some instances. Cambodia experience showed that strengthening policies for human resources including health insurance schemes improved health workers' motivation and attracted people to serve in the health sector. Sustained improvement in investment in midwifery training and numbers of midwives providing antenatal care and deliveries within an expanding primary health care network including a monetary incentive for facility-based midwives for every live birth conducted contributed to maternal mortality reduction in Cambodia (17). These combined investments motivated staff to provide quality care around the clock (20, 21). Formal engagement with NGOs and private sector improved availability of maternal and perinatal services but was affected by policy changes and resource withdrawal in Guatemala.

Local practice of continuous medical education and skill building was absent in low-resource settings. MCAT in Cambodia supported retention of skills and motivation. DRC adopted the strategy for engaging the ObGyn Congolese society for providing mentorship to providers for Emergency Obstetrical Care but the coverage has been low. Stakeholders emphasized on improving leadership and managerial skills at primary healthcare level. Private providers, often playing important roles in maternity care, were not always linked to the public system, and qualifications and supervision of providers varied widely. Reform efforts by the government improved accountability and consistency in qualification, certification and skills of private providers in Georgia. Stakeholders mentioned that engaging the private sector was critical to leverage their resources, healthcare workers, and other complementary services (e.g., diagnostics, digital services for linking data, referral transportation). In addition, many providers worked in both the public and private sectors.

Engagement of community members in the planning, designing, implementation and monitoring of health care services is crucial. Countries have demonstrated examples of how to strengthen community engagement, but those are yet to be implemented at scale. Sub-national level platforms existed to provide positive examples of linking communities with public health system in Cambodia, Guatemala, DRC and Pakistan. Such platforms and linkages to the public system were often not operationalised at scale. Community's engagement in maternal death reviews, sharing or decision-making was not common. Policies often included engagement of community for issues like client satisfaction but such policies were not adequately implemented. In some cases, community engagement referred to only engaging community health workers.

## Discussion

This series of case studies aimed to assess facilitators and barriers to improving maternal and perinatal survival and well-being in countries at different stages of the obstetric transition. The findings demonstrated that all countries made substantial improvements in maternal and perinatal health, although the rate of progress varied. Overall, the obstetric transition was a useful organizing framework to assess how countries at different stages achieved progress as well as provide an opportunity for cross-country learning. The findings underscored the importance of factors that are known (such as political commitment, supportive social policies, and functional health systems), and further highlighted new emerging areas that require additional attention to accelerate progress.

Many studies have demonstrated the importance of supportive social policies on reducing maternal mortality (22, 23). Societies that value female autonomy and empowering women have significantly higher life expectancies and lower infant mortality levels than those that do not (24). Country case studies that demonstrated rapid declines in mortality, such as Cambodia and Georgia, displayed a common approach in improving women's education and social staus as well as improving and sustaining skill levels of health workers. Countries with low female literacy rate over time showed high rates of gender-based violence, adolescent marriage and pregnancy, and persistent high burden of maternal deaths. The findings have demonstrated that it is essential to address social status of women and girls in all settings, regardless of stage of the obstetric transition.

Political leadership and commitment are also essential for continued progress. In 2007, Shiffman et al. published a framework for analyzing the determinants of political priority for global health initiatives (25). One of the elements listed in the framework was actor power—the strength of the individuals and organizations concerned with the issue. In a number of countries, political champions were very influential in advancing maternal health agenda but could not sustain their commitments after shifts in government leadership. National technical working groups were able to maintain momentum even during shifts in political leadership.

Policies and programmes are grounded in evidence-based recommendations. All countries in the case studies reported having updated national policies and standards based on global evidence-based guidelines and recommendations. However, the translation of these national policies and standards into large-scale programmes varied. The health service delivery system structure differs across countries, and global guidelines and recommendations need to be adapted to the context, with a focus on organization of levels of care including functional referral systems, available and competent health workers, and evidence-based intervention packages. The case studies highlighted the importance of providing guidance on how to adapt and implement global guidelines and recommendations into programmes within different contexts with the obstetric transition a useful framework.

In many countries, although the national policies and standards were included in national costed plans, the budget may or may not have been adequately allocated to successfully implement the plan. Stakeholders recommended that countries could benefit from having tracking mechanisms for monitoring implementation and resource disbursement as per the plan, both at national and sub-national or provincial level. In addition, the case studies demonstrated that the majority of women and families continue to pay significantly for out of pocket for health-related expenses. Several studies confirmed out-of-pocket expenditure can pose serious constraints to availing life-saving medical services (26–28). Large-scale donor funded programmes (or initiatives) providing pro-poor services could benefit from an exit or sustainability plan. The implementation capacity of the recipient government influences the time needed for the phase out (29). Governments can strategically plan budget allocation and increasingly pick up donor funded activities to sustain implementation. Stakeholders recommended that donors should strategically address gaps but not replace government resources to foster sustainability.

Even though national policies and standards were updated and adapted, there was often a gap in translating those the policies and standards into programmatic tools, such as, training curricula, job aids, etc., and moving to implementation. An evaluation in 2018 showed that adherence to clinical guidelines was low and in a systematic assessment across eight countries in sub-Saharan Africa, quality-adjusted (effective) coverage averaged 28% for antenatal care (30). In addition to strengthening the ongoing training, supervision, and mentorship, providing user-friendly practice tools and necessary equipment and supplies could help improve health workers to perform efficiently. Networks of care is a promising approach to strengthen relational aspects such as communication and team work among groups of health workers, but need adequate support to be sustained at scale as well as linkages with the community. WHO has defined a network of care for maternal and perinatal health as a collection of public and/or private health facilities and health workers deliberately interconnected to promote multi-disciplinary teamwork and collaborative learning to provide comprehensive, equitable, respectful, person-centered care from home/community to primary and tertiary levels (31, 32).

Across five of the six countries, the majority of maternal and perinatal services were provided at the primary and secondary level facilities, but the facilities often lacked adequate skilled health personnel. Stakeholders recommended empowering local authorities to identify gaps in health workers availability according to local needs (as opposed to a one-size national plan) to address the issues in a pragmatic way. Established and well-functional system for continuous medical education (CME) and in-service training are important prerequisites for retaining skills and provision of quality care. In some countries, an established midwifery cadre working within an enabling environment was successful at improving maternal and perinatal outcomes. For example, Cambodia implemented a successful strategy for rapid scale-up of midwives, including ensuring clarity around the role of midwives deployed at different levels of the health system, as well as promoting protection, autonomy and elevated social status to sustain motivation and strengthen quality of care.

There were a variety of emerging issues that are crucial for accelerating progress for maternal and perinatal health programmes. In these case studies, the role of the private sector emerged as well as the need to deliberately engage the private sector in maternal and perinatal care and services. In Georgia, a Stage IV country, strong government regulation of the private sector through adherence to quality service standards was a critical factor in reducing maternal mortality. WHO has developed a partnership framework for engaging the private sector, including for maternal and child health services (33). There are several examples of innovative partnership experiences between the public and private sectors for other countries to build upon to achieve universal health coverage (34).

Inequity continues to drive poor outcomes with the burden falling on vulnerable groups such as indigenous groups, ethnic minorities, displaced or migrant populations, urban slums, the poor, and others, varying by context. These disparities are not always routinely monitored, and the gaps in coverage of essential services were more pronounced in countries in stage III of the obstetric transition. For example, Guatemala has a large indigenous population who often faces cultural-linguistic challenges in receiving quality and respectful care and services. During the case study, national stakeholders highlighted targeted strategies to address these challenges and for an understanding of history and social structure to ensure equitable access to care (35). In Georgia, regionalization of maternal and newborn services was effective at reducing geographical disparities. While there are promising resources and innovations to improve access to services, including digital health/telemedicine, self-care and others, individual countries will need to identify and track the sub-national level vulnerable groups toward improving the health outcomes. This is a priority for future programmes.

In the case studies, there were gaps around engaging community and stakeholder partners in the design, implementation and monitoring of maternal and perinatal health programmes. There were examples of community-based platforms linked to the public health system that were effective at improving outcomes. The platforms could comprise of one community-based focal point who is responsible for maternal and newborn health including targeted engagement of vulnerable groups (e.g., women who are working in industry/factories, ethnic minority), local professional organizations representative(s), community organizations, local youth group members, and others. Models on community platforms for maternal and newborn health in various settings should be documented for sharing across countries (e.g., Rural Support Programmes' Network in Pakistan, COCODEs in Guatemala, etc.).

Another priority area is strengthening of data systems to capture real time information that can be used to improve programmes and track progress, including ensuring inequities are reduced. These data systems need to capture information from both public and private facilities and providers as relevant. WHO has developed guidance for maternal and newborn health indicators for inclusion in routine health information systems (Monitoring Maternal, Newborn and Child Health, 2011, https://www.who.int/publications/m/item/analysis-and-use-of-health-facility-data-guidance-for-rmncah-programme-managers). There are also tools to strengthen maternal and perinatal death reporting through maternal and perinatal death surveillance and response (https://www.who.int/publications/i/item/9789240036666) and quality of care measures for both provision and experience of care (Quality, Equity, Dignity, A Monitoring Framework for Network Countries, 2019. https://www.who.int/publications/m/item/quality-of-care-for-maternal-and-newborn--a-monitoring-framework-for-network-countries). As countries transition to digital data systems, there are opportunities for capturing individual level data throughout pregnancy and childbirth. In addition, there is a need to develop measures to capture maternal well-being and incorporate these measures into routine information systems. This was a gap in all countries, and a priority for the future.

### Strengths and limitations

Key challenegs in conducting the case studies included inadequate data for assessing maternal well-being as well as quality of care and use of private sector facilities. Data on the oraganization of MNH services at different levels of care was collected from national guidelines, no assessment or sampled survey was carried out to investigate the actual service delivery as this was beyond the scope. It is important to note that the case studies were conceptualized prior to the COVID-19 pandemic. Thus, the case studies did not explicitly include direct or indirect effects of the COVID-19 pandemic on maternal and perinatal health, although the delivery of health services during the pandemic was mentioned in some of the discussions that took place during data collection in 2020-2021.

## Conclusion

The country case studies were the first of its kind to take stock of how the policies and prorgammes evolved and influenced maternal health in country level. The six countries represented different WHO regions and various stages of the obstetric transition. While all six countries made remarkable progress in improving maternal and perinatal health, the pace of progress and the factors influencing the successes and challenges varied across the countries. The context, opportunities and challenges varied from country to country. Such variation does not allow to make one size fits all recommendations for countries at different stages of obstetric transition. Case studies on country-specific strategies could inform policies, planning programmes and strategic investment in countries with similar contexts. Broadly, it may be implied that in addition to improving programmes for quality services, non-health contextual elements are more important to prioritize for countries in all stages of obstetric transition for advancing maternal health agenda. Acceleration strategies for countries to achieve the targeted goals need to consider contextual elements, such as, women's social status, education and empowerment, political and geographic context, humanitarian and fragile situations etc. Countries in stage 3, who have already made progress in the non-health contextual elements, could benefit more by addressing the sub-national level gaps and disparities in programmes and services for effective coverage of essential interventions. WHO is currently updating the cut-offs for the obstetric transition and will develop a framework for programmatic guidance for settings at different stages of the transition. This will include lessons learned from the country case studies, as well as promote innovative solutions to optimize service delivery systems.

Based on these findings, WHO recommends two strategic directions for future maternal health programmes (see Box 4).

Box 4 **Strategic directions for maternal health programming.**

Based on the findings from the case studies, two strategic directions were identified for accelearting progress for maternal health programmes:

1. Implement and evaluate innovative service delivery models to accelerate reduction of mortality with a focus on high-burden settings using the obstetric transition as an organizing framework.

Promote translation of evidence-based guidelines and recommendations across different contexts using the obstetric transition as an organizing framework, linking with ongoing initiatives

Support innovative approaches to optimize functioning of service delivery including networks of care

Catalyze meaningful engagement of private sector through partnership models

Promote the crucial role of respectful and quality of care for provision and experience of care, including strengthening health worker education and training, assessment of competencies, mentorship and supervision with a focal on midwives

Promote scaling up effective and innovative medical products and commodities to address major causes of death and disability

Strengthen stakeholder and community engagement, empowering women for decision-making and active involvement of women, families and communities

Enhance routine information systems to improve real time tracking of quality of care and maternal outcomes

2.Expand our vision to address new realities with a focus on equity and well-being for all countries

Develop, refine and strengthen programmes to improve maternal well-being including reduction of morbidity

Promote a concerted effort to address preventing stillbirths including stigma.

## Author's note

The article is based on case studies focused on the evolution of country health and non-health policies, programme strategies and lessons in improving maternal health outcomes over the past two decades. The findings include the lessons drawn from six low-and-middle-income countries around various health and non-health contextual factors. Country policies and programmatic changes and prioritization approaches as well as delivery of services at different levels for maternal health changed over time and have influenced the progress. The article aims to provide directions to countries with similar contexts in accelerating progress in maternal, perinatal and newborn health and in achieving the desired goals.

## Author contributions

All authors listed have made a substantial, direct, and intellectual contribution to the work and approved it for publication.

## Conflict of interest

The authors declare that the research was conducted in the absence of any commercial or financial relationships that could be construed as a potential conflict of interest.

## Publisher's note

All claims expressed in this article are solely those of the authors and do not necessarily represent those of their affiliated organizations, or those of the publisher, the editors and the reviewers. Any product that may be evaluated in this article, or claim that may be made by its manufacturer, is not guaranteed or endorsed by the publisher.
