# Supplementary material for: Advancing maternal and perinatal health in low- and middle-income countries: A multi-country review of policies and programmes
Source: Front Glob Womens Health. 2022 Oct 10;3:909991. doi: 10.3389/fgwh.2022.909991 (PMC9589433; doi:10.3389/fgwh.2022.909991)
Supplement: Supplementary file 1 [file Table_1.DOCX]

**ANNEX**

**Findings on success factors, challenges and lessons learned**

The following tables provide a summary of the analysis from six case studies. Abbreviations used for countries are as follows, Cambodia-CAM, Democratic Republic of Congo-DRC, Georgia-GGA, Guatemala-GTM, Pakistan-PAK and Sierra Leone- SLE.

**Non-health context**

Social, environmental, political, gender and geographic factors that influenced the outcomes of the maternal and perinatal health were considered for this section.

| **Success factors** | **Challenges** | **Lesson learned** |
| --- | --- | --- |
| ***Status of women and girls*** | | |
| Strong laws and regulations for improving women’s and girl’s rights and education which accelerated reduction of early marriage and sexual and gender-based violence led to increased use of maternal health services (GGA, CAM) | Low levels of female education and High prevalence of female genital mutilation (SLE).  Women’s and girl’s rights and status varied at sub-national level, i.e., among low performing provinces, indigenous population etc. (GTM, PAK)  High rates of adolescent pregnancy and birth (DRC, SLE, GTM). | Countries with strong laws and regulations for improving women’s and girls’ rights and education showed reduction in the proportion of adolescent pregnancy and sexual and gender-based violence and increase use of maternal health services.  Women’s and girls’ status varied at provincial level, especially in countries in stage III of the obstetric transition. |
| ***Emergency Response and Resilience*** | | |
| Humanitarian crisis, natural disasters and epidemics drew global attention and funding (SLE, DRC, CAM, PAK). | Natural disasters, humanitarian crises significantly affected service provision and uptake.  Resource allocation suffered from ad hoc and/or short-term planning (SLE, PAK, DRC, GTM). | Emergencies hampered routine care and displaced people in many instances.  For a number of years following the disasters, countries face constant sub-national level migration of displaced populations. However, maternal and perinatal health are not always prioritized at the service delivery level. |

**Policy and System**

The following factors were included in this section: 1) national/sub-national policies, strategies and plans; 2) national targets; 3) leadership; and 4) financing.

| **Success factors** | **Challenges** | **Lesson learned** |
| --- | --- | --- |
| ***National and sub-national policies, strategies and plans*** | | |
| Prompt and continuous adoption of global guidelines and standards (all)  Maternal health incorporated in the development agenda and non-health policies. (GGA, GTM)  Alignment between national plans across various sectors contributed to improved health outcomes (CAM)  Global/regional advocacy/events influenced national commitment (DRC, SLE, PAK)  Strong local stewardship is important (PAK) | Sub-national variation in policy uptake (PAK)  Major policy shifts with Political changes (GTM)  Weak policies and/or regulation for girls/women’s rights and protection (DRC, SLE, PAK).  Weak regulation and implementation of policies for the indigenous girls and women (GTM).  Private sector is not consistently engaged in policy adoption and planning for public health services (all except GGA) | Plans were updated promptly to include evidence-based guidelines, standards, and interventions but their implementation was delayed mainly because plans were not consistently costed and made adopted at sub-national level.  In devolved systems, adoption/ adaptation of policies and standards was largely driven by the sub-national leaders/authorities. Provincial/regional level sensitisation influenced adoption, resource allocation and roll out.  Global/ regional events reinforced commitment (e.g., DRC’s commitment to Abuja Declaration in 2001, SLE’s participation in QED network). However, policy and programme implementation was linked to resource allocation at sub-national level. |
| ***Maternal and Newborn Targets*** | | |
| All the countries had national targets for maternal mortality and neonatal mortality.    The targets often coincided with the planning cycle (CAM, GTM, SLE, DRC) | Targets for stillbirth rate were missing in 3 of 6 countries (CAM, GTM, DRC). | Global targets helped countries to establish national targets.  Countries set and revised targets during the development of the health sector plans and/or national development plans.  Maternal and neonatal mortality targets are more common than stillbirth targets. |
| ***Leadership*** | | |
| Sustained political will over a long period of time can improve maternal and newborn health (CAM, SLE, GGA)  High level champions (President/First Lady/Parliamentarians), and National MNH working groups are essential (CAM, GTM, PAK, SLE, DRC) | Political shifts impacted service delivery and resource allocation (GTM, PAK)  Sustaining engagement of champions with shifts in government (GTM) | Having sustained political will helps to develop/reinforce comprehensive policies and maintain the momentum over time.  Progress is stalled when key leaders are frequently changed. National policies and implementation strategies get substantially and frequently modified with the change of the political government. The national technical working group can support maintaining momentum even with shift of political leadership.  Political champions are very effective but not sustainable with change in government. |
| ***Financing*** | | |
| Sustained government financing in maternal and newborn health promoted consistent progress.There was significant reduction in out-of-pocket spending ( GGA, a Stage IV country) | Low levels of total health expenditure for maternal and perinatal health (DRC, CAM, GTM).  Large scale programs dependent on donors (PAK, SLE, DRC). Distribution of donor resources not well prioritized (SLE)  Plans are inadequately funded (PAK, SLE, DRC, CAM, GTM)  Significant out of pocket spending. (all)  Innovative financial schemes are often supported by external donors (SLE, DRC, PAK, CAM) | Comparison shows major declines in out-of-pocket (OOP) spending in health in some countries but still about half of all health expenditure across countries. Large-scale programmes, often supported by external donors, are time-bound and resource allocation suffered from withdrawal of donor support. |

**Programme Content**

Programme content includes the organization of the system and health care services as described in national guidelines including networks of health care facilities and providers.

| **Success factors** | **Challenges** | **Lesson learned** |
| --- | --- | --- |
| ***System of care*** | | |
| Clear/structured levels of services for maternal health care (ANC, birth, PNC) and type of provider (all) | First levels of care are overburdened; providers have limited competencies and lack equipment. There are weak referral systems and limited linkages to community levels (GTM, CAM, PAK, SLE).  Definition of levels of services varies across countries (all)  Limited management and leadership skill of some health managers (CAM) | Definitions of levels of care are country-specific. Therefore, the content for each level (infrastructure, human resources, services, readiness etc.) do not always follow global definitions.  Linkages for referrals and/or with non-communicable/infectious disease is not strong. Women/families are required to move between different facilities for care. |
| ***Maternal and Perinatal Services*** | | |
| Guidelines and protocols are updated regularly for maternal health (all)  Government oversight and engagement with private sector improved care (GGA)  Institutionalisation of standardised care (Effective Perinatal Care) significantly improved perinatal outcomes (GGA) | Trained providers could not/did not always follow standards (CAM, PAK, SLE, GTM)  Private sector is often unregulated. Issues with compliance, coordinating, linkages with private sector (PAK, SLE, CAM, GGA)  Leadership and Management course/training for health care workers to perform as better managers is non-existent (PAK). | Adopting/updating technical guidelines, protocols or training curriculum did not always lead to evidence-based practices in provision of quality and respectful care, especially in high volume facilities.  Guidelines for maternal and newborn health sometimes not consistent across levels of care.  Management of morbidity is sometimes inadequate and/or inconsistent in the protocols. Some important morbidities, such as infections, anaemia, fistula and postpartum depression, are not included. |
| ***Networks of care*** | | |
| Networks of care facilitated maintenance of providers- skills and supervision especially the midwife coordination alliance team (MCAT) (CAM).  Local health committees (COCODEs, and networks of community health facilitators and traditional birth attendants) served as important platform for the community (GTM). | Local health networks at the community level were not linked to the formal system and did not sustain over time (GTM).  Lack of funding to regularly organize MCAT meetings (CAM). | Networks were established and piloted across settings but often not formalised for implementation at scale.  Some networks are operational and working in parallel and not integrating with each other – implying lack of coordination and awareness – limiting the right information or disseminating to all facilities. |

**Programme Implementation**

The components under Programme Implementation include access to services, quality of services, data systems, and human resources.

| **Success factors** | **Challenges** | **Lesson learned** |
| --- | --- | --- |
| ***Access to services*** | | |
| Sub-national level targeted strategies reduced disparities in provinces/ regions*. (PAK, CAM, SLE, GTM)  Various pro-poor financing models were piloted. (PAK, CAM, SLE)  Financial incentives contributed to an increase in facility-based birth (CAM)  Access to services improved with extension of care through local NGOs (GTM).  Digital/ telemedicine (WhatsApp, toll-free help line, etc) could improve access to services among vulnerable groups | Disparities continue across provinces/municipalities and among indigenous populations. (PAK, CAM, GTM, SLE, DRC).  Primary level childbirth facilities are not equitably located, especially in rural areas (all except GGA)  Use of postnatal care for women and newborns is lower than coverage for antenatal and childbirth care (GTM, GGA, DRC) | Surveys demonstrated in-country disparities across various population groups, provinces, and income groups. Inequity in services is often not reported routinely.  Financing schemes to improve access to the poor were often at small scale and/or donor dependent, as noted under financing. Such schemes were not feasible to sustain in the long run without government budget allocation.  Sub-national or local plans are not adequately developed to address disparities at districts or local communities.  WHO guidelines on self-care could support improving access. |
| *Examples: Extension of services by NGOs in rural Guatemala; Provincial Health Commissions in Pakistan; Perinatal care regionalization reform and regional distribution of perinatal care services in Georgia. | | |
| ***Quality of care*** | | |
| Evidence-based policies, guidelines and standards in place (GGA, CAM, PAK, SLE)  Quality of care improved through perinatal care regionalization reform in GGA.  Being part of the global Quality of Care Network supported quality of care implementation (SLE)  Providers trained in quality and respectful care had improved confidence and skills (GGA, CAM)  Providers’ network (MCAT in Cambodia) helped to support midwives to deliver quality services. | Limited or no quality of care indicators included or reported in routine health information systems around provision and experience of care (all but GGA)  Maternal death surveillance and response is implemented but data is often not integrated and used in routine data systems.  Limited regulations and adherence to national guidelines/protocols for private sector providers/facilities (all but GGA)  Poor quality of services demonstrated (SLE, PAK, GTM)  Increasing caesarean section rates especially among the rich and in private sector (all)  Inadequate training and supervision for providers on quality and respectful care (PAK, SLE)  Lack of morbidity data to understand care-seeking and management. (all) | Adoption of policies are necessary but not sufficient to improve quality of care outcomes. Most countries have recently adopted policies for improving quality of care but yet to be institutionalised.  Training of health workers to improve quality of care has taken place in most countries but has seen varying levels of success based on available data.  A supportive enabling environment is essential to enable health providers offer quality services.  Providers are overburdened and this has implications for poor quality of services.  All countries have had increased CS rates, especially among the wealthy quintiles.  Formal networks of providers helped to support providers in retaining skills and motivation.  Strong regulation and monitoring of the private sector by the government helped improve quality of care.  National programmes need to strengthen focus on maternal and neonatal morbidity including anaemia, obstetric fistula, mental health and others and incorporate into programs. |
| ***Data*** | | |
| Transitioning to digitalised data systems helped access to real time data, such as DHIS2 (GGA, DRC)  A real-time electronic maternal and child health management information system allowed continuous monitoring of pregnant women and newborns (GGA)  Sub-national level data availability and improved capacity in data use improved planning and designing efficient strategies at local level (CAM, DRC)  Having routine data record and report on morbidities helped improve preparedness and management at facilities (GGA). | Limited/no routine data for programme and health system planning around Quality of Care and equity (CAM, GTM, PAK, SLE, DRC).  Information on prevalent morbidities (e.g., anaemia, hypertension, depression) are either absent or incomplete in routine data systems. (All)  Routine data systems often do not include data from private providers or private facilities. (CAM, GTM, PAK, SLE, DRC).  Civil registration and vital statistics systems (CRVS) often weak and varies across sub-national areas. (All)  Inadequate (or absence of) routine data and reporting on maternal and newborn morbidity (exceptions – GGA, DRC). | Data availability and use at the local level enabled local decision makers, including local government authorities, to address gaps.  There were limitations in routine data for morbidities, quality of care, and equity, as well as information from private sector.  Most of the countries experienced challenges with birth and death registration.  Transitioning to digitalised data systems helped improve data availability and use at the local level in two countries. Access to real-time data from pregnancy through childbirth and postpartum period and allowed continuous monitoring and assessment of the quality of care.  Adoption of the MDSR guideline to national programme at scale showed improvement in reporting and investigating the causes of deaths. However, response mechanism by facilities is yet not well established. |
| ***Health care workers*** | | |
| Strengthened policies for human resources including health insurance schemes improved health worker motivation and attracted people to serve in the health sector (CAM).  Competence of health professionals improved significantly with introduction of Continuous Medical Education for perinatal care providers (GGA).  Reform efforts by the government improved accountability and consistency in qualification/ certification and skills of private providers (GGA). | Distribution of health workers is often not equitable. Deploying and retaining female providers can be challenging in rural areas (CAM, PAK, GTM, DRC, SLE).    Inadequate training and supervision for maternal and perinatal health providers (all but GGA).  Local challenges include language barriers, cultural differences, unclear roles and inadequate distribution of human resources providing care for diverse populations (such as, indigenous, tribal, etc.) (GTM, PAK).  Private providers are not linked to the public system and qualifications, supervision of providers vary widely (all but GGA). | Policies and regulations elevating the status of health workers, especially serving at the primary and community levels, improved motivation and performance.  Formal engagement with private sector improved availability of maternal and perinatal services but was affected by policy changes and lack of accountability.  Local practice of continuous medical education and skill building is absent in low-resource settings. |
| ICM standards are increasingly adopted for midwifery training (all).  Formal support system improved motivation and performance (CAM). | In-service training, supervision and on-the job refreshers do not often follow the national guidelines and standards or are absent (GTM, PAK, SLE, DRC).  There is a shortage of skilled midwives. Their roles are not clear at service delivery points and they face discrimination (GTM, PAK).  Midwives do not have a clear role in management of childbirth (GGA). | Adopted midwife-led care policies were not evident in practice.  Definitions and training varied across countries for different cadres of maternal and newborn health providers (e.g., nurse-midwives, community midwives, trained traditional midwives etc.).  Deployed midwives were not recognised or could not perform their roles in some instances. |
| ***Private Sector*** | | |
| Strong regulation for certification and improved accountability as well as engaging private sector in planning improved policy and protocol adherence and improved perinatal outcomes. (GGA) | Weak regulation/ rules of hospital licensing resulted in uncontrolled proliferation of medical facilities with questionable quality (all). | Private sector has been playing an important role in maternal and newborn health but is not often linked or engaged with the formal system. Strengthened regulations improved training, certification and compliance. |
| ***Community Engagement*** | | |
| Sub-national level platforms provide positive examples of linking communities with public health system. (GTM, PAK, CAM, DRC).  Engaging local professional organization representatives was helpful to improve service delivery (PAK).  In DRC, Community is formally linked to the health system and its role is well defined even in decision-making at any level of the health system. but not always operational. | Community platforms and linkages to the public system are often not operationalised at scale (all).  Community’s engagement in maternal death reviews, sharing or decision-making is not common. (all) | System/platform for engaging communities in planning, sharing, reviews (including death reviews) etc., is weak, informal or absent. Policies often include engagement of community for issues like client satisfaction but such policies are not adequately implemented.  Communities often do not take part in planning of health services and strategies if not linked formally to the health system. In some cases, community engagement referred to only engaging community health workers.  Sub-national platforms are one mechanism to strengthen for improving community engagement. |
