# Supplementary material for: Advancing maternal and perinatal health in low- and middle-income countries: A multi-country review of policies and programmes
Source: Front Glob Womens Health. 2022 Oct 10;3:909991. doi: 10.3389/fgwh.2022.909991 (PMC9589433; doi:10.3389/fgwh.2022.909991)
Supplement: Supplementary file 2 [file Table_2.DOCX]

**Annex II: Key factors enabling and challenging policies and programmes in each country.**

| **Country** | | **Georgia** | | **Cambodia** | **Guatemala** | **Pakistan** | **Sierra Leone** | **DRC** |  |
| --- | --- | --- | --- | --- | --- | --- | --- | --- | --- |
| **A. Context (non-health), policies, governance, leadership, financing** | | | | | | | | |  |
| **Policies**  **(Health and non-health policies, political will)***  *not measuring implementation | | Sustained political will in advancing maternal and perinatal health | Sustained political will in improving the status of women, education, infrastructure.  National Day for MNH observed. | | Laws and policies evolved in favour of women including addressing the inter-cultural barriers.  Political changes affected health strategies. | Continuous improvement of MNH plans and technical resources (EPHS and MSDS).  National Health Vision and UHC agenda prioritized by all provincial and federal governments. However, the momentum varies across provinces. | Strong political will and prompt adoption of evidence-based policies and global standards. | Strengthened commitment to Abuja Declaration (2001) and coordination by DSFGS/MoH. |  |
| **Leadership** | | Continued leadership of Georgian government in strategic reform of the health system, engagement and regulation of private sector and revitalization of universal health coverage (UHC). | First lady (Samdach Kittiprittbandit Bun Rany Hun Sen) played a pivotal role;  Working group at ministry and program level to coordinate support. | | Important lessons on working with Congress women;  Observatory for Sexual and Reproductive Health (OSAR) sustained to advance MH agenda. | Provincial and federal leadership complemented by Healthcare Commissions helped advance maternal health. Commitment from President and Prime Minister to address population issues. | Strong leadership by government and political commitment to address Gender-based Violence.  Champions at the highest level (CARMMA campaign) | Consensus around the SRMNEA-Nut platform (including the Ministry of Health and its specialized programs, partners). |  |
| **Financing**  *(includes NHA data)* | | Rapid increase in investment in health. UHC with minimal Basic Benefit Package strengthened financial protection, reduced out-of-pocket (OOP) payments from 81% in 2000 to 55% in 2017. | Robust economic growth. Total Health Expenditure: 6% of GDP.  Government increasingly taking up donor funded activities; | | Budget allocation increased over time but inadequate funding levels remain a major problem for public health expenditures. | Large donor funded programmes; government health expenditure (% of GDP) is showing increasing trend yet high OOP spending. | Large donor funded programmes (low domestic funding); During epidemics/ war, distribution of resources was not well prioritized to build resilient health systems; Very high per capita total health expenditure, OOP spending in health was 44.7% in 2018. | Health sector is largely external donor funded. Still inadequate.  THE 0.5% of GDP. |  |
|  |  |  | Cambodia national health accounts (2012-2016) health expenditure report: little reduction of out-of-pocket expense, from 61.2% in 2012 to 60.4% in 2016.  <https://iris.wpro.who.int/handle/10665.1/14362> | |  |  |  | OOP spending for health reduced but still high (42%). |  |
| **B. Programme and service level factors** | | | | | | | | |  |
| **Financing schemes**  (Policy and project based) | | Not applicable | Scale – national but suboptimal coverage.  Innovative financing schemes piloted to inform scaling up to reduce financial barriers- National health insurance (NSSF), Cash support programs to women and children <2 years old of family with HEF card or priority card. Sub-decree No.88: Financial scheme for people with poverty and children under 5 affected by covid-19. | | No financing schemes | Scale - national  Various pro-poor schemes undertaken including Benazir Income Support Programme, Ehsaas programme and Sehat Sahulat programme but still did not cover the entire population. A pilot on Outpatient Department health insurance using capitation model is currently underway 4 union councils in Islamabad Capital Territory. | Scale - national  Free Health Care Initiative (FHCI initiated in 2010) improved access to health service by lowering financial barriers but is donor dependent. | Mutual Health organisations (MHOs) aim to improving healthcare quality while minimising expenses. But weakly executed and not empowered to influence. |  |
| **Private sector** | | Fully served by the private sector. Initially largely deregulated, currently – strengthened accountability and regulation.  Domestic Private Health Expenditure (PVT-D) as % Current Health Expenditure (CHE)^[[1]](#footnote-1)^: 60% | Issues of compliance, poor links with public sector.  Domestic Private Health Expenditure (PVT-D) as % Current Health Expenditure (CHE): 58% | | Private sector generally does not serve in rural areas.  Inadequate coordination among MSPAS, IGSS & private providers.  Domestic Private Health Expenditure (PVT-D) as % Current Health Expenditure (CHE): 52% | Private providers are the major providers for MNH but are rarely engaged in planning or coordination. Issues of compliance.  Domestic Private Health Expenditure (PVT-D) as % Current Health Expenditure (CHE): 64% | There are a few private sector providers although they do not play a major role in MNH healthcare.  Domestic Private Health Expenditure (PVT-D) as % Current Health Expenditure (CHE): 64% | The private sector is regulated (taken into account in the general regulation norms) but they don’t often follow the rules (reporting, etc.)  Domestic Private Health Expenditure (PVT-D) as % Current Health Expenditure (CHE): 50% | |
| **Linkages/networks** | | Regionalization of perinatal care service delivery implemented, that strengthened linkages between health care facilities of different level. | Networks of health facilities established (district model from 1995) to expand access to overall health care. | | Local health committees, COCODEs, and networks of community health facilitators and traditional birth attendants, constitute important elements of the country’s health system that have not thus far been successfully linked to the national health system. | Healthcare delivery infrastructure is complemented by a large network of community health workforce (LHWs, FWWs, vaccinators etc.) besides a widespread private sector healthcare delivery system. Various public private partnership models adopted. Yet to reach the desired scale. | Not applicable | Network of health facilities established (referral system) but faces difficulties in operationalising at scale. |  |
| **Facilities for childbirth** | | The infrastructure and human resource capacities strengthened for childbirth facilities. | MPA and CPA1 are preferred but EmONC readiness vary across provinces. | | CAPs and CAIMIs provide the major MNH services. Preparedness varied by geography. | Doctors, lady health visitors (LHVs) and community midwives (CMWs) provide MNH services at PHC level. However, private providers are preferred. | PHUs are preferred but often not ready and lacks efficient referral mechanism. |  |  |
| **Morbidity** | | Perinatal morbidity and mortality reduced due to the improved perinatal services, institutionalization of evidence-based effective perinatal care and regionalization of perinatal care services. | High prevalence of anaemia in women continued over the decades while haemorrhage and hypertension are major causes of maternal deaths. Low birthweight remained at 7% since 2010. | | Morbidity data only available in annual reports. Inadequate routine data to assess trends and address gaps. | Maternal morbidity data is increasingly available in the DHS and maternal mortality survey 2019. Ongoing dialogue on strengthening maternal morbidity data in the routine health information system. Facility records can be incomplete and not linked. | Inadequate preparedness at PHU level and low diagnostic and management facilities for morbidities.  Inadequate routine data on morbidity. | DHIS2 collects morbidity data.  Although DHIS2 is implemented nationally, data is not routinely used to check trends and prevalence in various provinces. |  |
| **Quality & respectful care** | **Policy/ guideline** | Improved quality of perinatal care by strengthening licensing requirements, implementation of regionalization principles, strengthening human resource capacity, introducing perinatal audit, introducing quality indicators, introducing selective contracting. | Strengthened technical guidelines. | | Strengthened policy. | WHO guidelines and standards have been adapted in the country. Various QoC tools (i.e., Robson TGCS, MPDSR, EENC, SCC etc) have been introduced, however, the scale is limited. Health Care Commissions at provincial level increasingly adopting Respectful Maternity Care. | Policy, guidelines updated. BPEHS includes the quality standards but not institutionalised.  Part of QED Network. | Various evidence-based interventions were adopted but QED or quality in provision and experience of care is not evident. Increasing trend in CS. |  |
|  | **Training** | CME reintroduced for perinatal care providers. They are mandated for participation in regular capacity building trainings. | The pre-service curriculum partially met the ICM Standards. The competency-based Education for Midwives is needed to be strengthened. | | Upgraded curricula but the Trained Midwives are not always receiving ICM standard training. | Updated guidelines are sporadically used for in-service capacity building. Poor quality of care and abuse is still a challenge. Need for strengthening the pre-service and in-service capacity building for all cadres. | Not trained yet | Inadequate training. Poor quality of care and evidence of abuse. |  |
|  | **data** | Perinatal data quality and completeness improved with introduction of the National Birth Registry | Quality and experience of care not routinely recorded | | Quality and performance indicators not recorded or reported. | Lack of data. MPDSR use is limited. Quality and experience of care not routinely recorded – only captured in small scale surveys and studies. | Lack of data on quality and respectful care | MPDSR is reported in DHIS2 (Number of cases and number of reviews) but not often used for decision-making at lower level. |  |
| **Equitable access for vulnerable** | **coverage** | Introduction of UHC helped to improve access to services | Steady increase in coverage.  Access to BEmONC and CEmONC varied across provinces.  FTIRM (2016-2020) planned - at least 4 BEmONC and 1 CEmONC facility per 500,000 population. | | Inequity in coverage increased after withdrawal of extended service by NGOs (indigenous). Access varies for mother and newborns. | Disparities across provinces and socioeconomic groups.  Various models were adopted towards improving access to BEmONC and CEmONC services but the reach is not universal. UHC Benefit Package is planned for implementation in 40 priority districts in two phases. | Surveys show high coverage of essential interventions.  Delays in accessing timely and appropriate care due to a weak referral system. | Provincial disparities.  Wide gap between services among the poor and the rich.  Gaps in continuum of care for mothers and newborns. |  |
|  | **Care-seeking** | High ANC coverage  Almost 100% institutional deliveries  Yet, only 47% mothers received a postnatal check up | Health Centers (HC) and CPA1 District Hospitals are the major providers of maternal health care in public system;  HCs are frequently many miles away from small villages. | | CAPs and CAIMIs are not equitably located in rural areas. | Informal and private providers are preferred providers. Preference for directly seeking care at referral or tertiary care centres.  Increasing CS rates. | PHUs are main provider for MNH but often not ready to provide the quality services | Increasing CS rates among the rich. |  |
| **Data** | **Routine monitoring** | Electronic Module for Pregnant and Newborn Health Surveillance (National Birth Registry) in 2016 helped to improve availability of data. | Sub-national level Commune Database improved data use and designing efficient strategies at local level. Data quality scorecards helped to identify gaps in recording and reporting. | | Inadequate routine data to monitor QoC, morbidities, disparities across, age, location, SE groups. | DHIS is in place and tertiary facilities maintain independent data.  Inadequate routine data on QoC, morbidity and disparities. MPDSR coverage is very low. Electronic medical record systems sporadically adapted at tertiary care level in both public and private sector. A pilot on use of electronic data management at PHC level is underway. | HMIS data not disaggregated for routine reporting. Inadequate data on hard-to-reach areas.  MDSR is challenged by underreporting, data quality, inadequate system integration. | Système National d’information sanitaire (SNIS) 2 data used for review and decision making but disaggregated data (adolescent age ranges, location etc.) not analysed and used by providers at local level |  |
|  |  |  | Routine HMIS data not often disaggregated to assess quality, equity in access. | |  |  |  |  |  |
|  | **Registration systems, surveys** | Gaps in maternal death registration system (RAMOS 2006) were addressed over the years by improved surveillance, mandatory maternal mortality reporting etc., | CRVS assessments helped to improve registration. | | Birth and death registration improved over time. Annual reports include morbidity and equity data but inadequate data on quality of care. | Birth registration is low.  Reporting of deaths at community and facility level is not streamlined. | Need for strengthening birth and death registration and reporting systems. | Low registration of birth and deaths. |  |
| **Response in emergencies** | | During emergencies and epidemics perinatal services are maintained, not disrupted | HEF for Covid 19. There are long-term response strategies for provincial level emergencies. However, there is a need for continuous support to update and implement as per the local needs. | |  | Routine services are disrupted during emergencies. Lack of consistent strategies for resilient response despite prioritization of mothers and children in all humanitarian response plans. | Disrupted service during emergencies and epidemics. Distribution of resources were not well prioritized depriving maternal health services significantly. | Policies and strategies related to management of outbreaks have been developed by the MOH and are being regularly updated. Resources are inadequately allocated to manage displaced people. |  |
| **Human resources** | **General** | Specialist doctors provide MNH care. Continuing medical education system revitalised for obstetric and neonatal health professionals through perinatal care regionalization reform. | Private sector and informal providers account for 61% and 26%, of all service provision respectively. Regulation of private practice professionals is reportedly challenging. | | 33% non-SBAs deliver at home. Auxiliary Nurses, traditional midwives and doctors remain the major providers for MNH. The implementation of intercultural policy for MH is inconsistent. Dearth of providers speaking indigenous language. | Private providers cater to majority of all deliveries in the country. Provincial disparities in SBA. Vacant positions of female providers, especially at community, primary and secondary level. Inequitable distribution – concentration of providers in urban centers. | HRH-2-6.4 per 10,000 population;  MNH workforce lack motivation, supportive supervisory and mentoring support for skill building and retention.  Lack of adequate training and continuing education in quality and respectful care of various MNH providers at all level. | Nurses and midwives (74,7%) attend birth.  The Midwives education system is established and has started producing health workforces. |  |
|  |  |  | health insurance coverage to formal sector workers (under NSSF). | |  |  |  |  |  |
|  | **Midwives** | In contrast with excessive number of obstetrics and gynaecology specialists, the shortage of midwives pronounced, their recognition, status and roles limited | Royal Decree establishing Cambodian Midwives Council (2006) and subsequent efforts in deployment of midwives improved access to maternity care.  Quarterly Midwife Coordination Alliance Team (MCAT) meetings for supervision. | | Roles of midwives suffered from lack of clarity and discrimination. “Traditional midwives” are trained and included in the public system. However, they do not comply with the global definition of “skilled provider.” Traditional midwives, main providers in rural areas, are not part of the public health system but there is increasing coordination. | Midwife-led care is yet to be accepted and practiced.  Midwifery capacities are not in line with ICM standards. Efforts are ongoing to update the midwifery training and competencies. | 70% deployed midwives are in urban areas serving 38% of the population. Inadequate training using ICM standards. | As per policy, 1 for 5000 population and to be trained using ICM standards.  Serving in the Health Centers and referral hospitals.  4270 midwives and nurses (graduates). Work underway on their deployment and coverage by MOH. |  |

1. Share of current health expenditures funded from private sources; NHA 2018 data. [↑](#footnote-ref-1)
